# BIN1 favors the spreading of Tau via extracellular vesicles

**DOI:** 10.1038/s41598-019-45676-0

**Published:** 2019-07-01

**Authors:** Andrea Crotti, Hameetha Rajamohamend Sait, Kathleen M. McAvoy, Karol Estrada, Ayla Ergun, Suzanne Szak, Galina Marsh, Luke Jandreski, Michael Peterson, Taylor L. Reynolds, Isin Dalkilic-Liddle, Andrew Cameron, Ellen Cahir-McFarland, Richard M. Ransohoff

**Affiliations:** 1 Biogen, 225 Binney St., Cambridge, MA 02142 USA; 2 Fulcrum Therapeutics, 26 Landsdowne St, Cambridge, MA 02139 USA; 3 Third Rock Ventures, 29 Newbury Street, Suite 30, Boston, MA 02116 USA; 4 Present Address: Astellas, 1030 Massachusetts Avenue, Cambridge, MA 02138 USA

**Keywords:** Microglia, Alzheimer's disease

## Abstract

Despite *Bridging INtegrator* 1 (*BIN1*) being the second most statistically-significant locus associated to Late Onset Alzheimer’s Disease, its role in disease pathogenesis remains to be clarified. As reports suggest a link between BIN1, Tau and extracellular vesicles, we investigated whether BIN1 could affect Tau spreading via exosomes secretion. We observed that BIN1-associated Tau-containing extracellular vesicles purified from cerebrospinal fluid of AD-affected individuals are seeding-competent. We showed that BIN1 over-expression promotes the release of Tau via extracellular vesicles *in vitro* as well as exacerbation of Tau pathology *in vivo* in PS19 mice. Genetic deletion of *Bin1* from microglia resulted in reduction of Tau secretion via extracellular vesicles *in vitro*, and in decrease of Tau spreading *in vivo* in male, but not female, mice, in the context of PS19 background. Interestingly, ablation of Bin1 in microglia of male mice resulted in significant reduction in the expression of heat-shock proteins, previously implicated in Tau proteostasis. These observations suggest that BIN1 could contribute to the progression of AD-related Tau pathology by altering Tau clearance and promoting release of Tau-enriched extracellular vesicles by microglia.

## Introduction

Alzheimer’s Disease (AD) is a complex, multifactorial neurodegenerative disorder leading to dementia, primarily in the elderly and currently affecting an estimated 5.7 million Americans (www.alz.org). AD is characterized by two specific type of lesions of the brain: extracellular senile plaques, composed of aggregated amyloid-β (Aβ) peptides along with other components, and intraneuronal Neurofibrillary Tangles (NFT), containing hyper-phosphorylated misfolded Tau^1,2^. While amyloid deposition could be found in the basal portions of frontal, temporal and occipital lobe^1^, NFTs appear originally in the transentorhinal region, and then in the entorhinal cortex, at a stage of neuropathological progression defined as Braak’s Stages I and II^1^. Subsequently in stages III and IV, NFTs are found in CA1 region, and then subiculum, CA4 region, amygdala, putamen and thalamic nucleus^1^. Finally, in stages V and VI, pathological Tau can be found throughout the hippocampus, accompanied by widespread neuronal loss^1^. In the last few years, it has been proposed that progressive Tau pathology may be associated with “prion-like” spreading between brain regions which are functionally connected^3^. In this view, hyper-phosphorylated and misfolded Tau aggregates give rise to conformation-dependent “seeds” that promote a templated propagation of intracellular aggregated deposits. Following their intercellular transfer, these aggregates induce similar aggregation in the receiving cells. Several mechanisms of Tau intercellular seeding have been proposed, including release and uptake of free-floating Tau aggregates or fibrils^4,5^, trans-synaptic transfer of Tau aggregates between neurons^6,7^, tunneling nanotubules^8^ and transfer via extracellular vesicles such as exosomes^9–13^ as well as ectosomes^14^.

Extracellular Vesicles (EVs) are membranous vesicles composed of a lipid bilayer and containing proteins and RNAs characteristic of the cell of origin. Exosomes comprise a subset of EVs ranging between 30–150 nm in diameter, originating in the endosomal compartment^15^. Exosomes represent a means of waste disposal for cells as well as a form of long-distance intercellular communication^15^. The Endosomal Sorting Complex Required for Transport (ESCRT) mediates active release of exosomes by every cell type^15^, including all categories of Central Nervous System (CNS) cell^16–18^. Exosomes are present in body fluids including Cerebrospinal Fluid (CSF)^19^, blood^20^, and urine^21^, facilitating their analysis and investigation.

Genome-Wide Association Studies (GWAS) have identified several Single Nucleotide Polymorphisms (SNPs) strongly associated to increased risk of developing Late Onset Alzheimer’s Disease (LOAD)^22,23^. Several of these SNPs have been found in loci near genes that encode for proteins involved in endosomal recycling such as BIN1, CLU, PICALM, RIN3, CD2AP^24,25^. SNPs in the locus harboring *Bridging INtegrator* 1 (BIN1) gene show the strongest association with AD, after Apolipoprotein E (*APOE*)^23^.

A member of the Bin/Amphiphysin/Rvs (BAR) family of adaptor proteins that regulates lipid membrane dynamics^26,27^, *BIN1* is ubiquitously expressed throughout the body, with the highest expression in skeletal muscle^28^, followed by the brain^29,30^. BIN1 is involved in a wide range of cellular functions associated with membrane curvature, including phagocytosis and clathrin-mediated endocytosis^26,27,31,32^.The genetic association between *BIN1* and AD prompted examination of the role of BIN1 in the CNS. In the brain, *BIN1* is expressed in multiple isoforms: the ubiquitously expressed isoforms 9 and 10, together with brain-specific isoforms 1 through 7^26,27,33^. Recently, it has been shown that BIN1-containing extracellular vesicles are released by cardiomyocytes in the blood stream via ESCRT-III, raising the possibility that analogous events might be associated with BIN1 action in the brain^34^.

Where sufficient data are available to establish genotype-phenotype relationships, the vast majority of AD-associated genetic variants are related to the phenotype of amyloid deposition. *BIN1*-associated SNPs are quite distinct, being significantly associated with the level of total Tau and pTau in the CSF, but not with Aβ^35^. Furthermore, variant rs59335482 is associated with increased *BIN1* expression and Tau loads, but not with Aβ loads in AD brains^36^. Interestingly, increased expression of *BIN1* mRNA and protein have been observed in post-mortem brain samples from AD-affected individuals^36–38^. Bin1 levels positively correlated with NFTs, but showed no correlation with diffuse neuritic plaques or with the amount of Aβ in the brain, regardless of genotype^36–38^. These data suggest that BIN1 is likely involved in AD as a modulator of Tau pathology, rather than as a promoter of Aβ deposition. Of note, BIN1 has been reported to interact with Tau in mice^36^, and had been previously implicated in intracellular endosomal trafficking of Tau^7^. It remains unknown whether *BIN1* gene variants contribute to neurological disorders associated to mutant forms of Tau itself. Therefore, understanding how BIN1 promotes Tau pathology in AD may provide unique insight into the commonest form of non-genetic Tau pathology.

Here, we report that BIN1 and Tau are present in seeding-competent EVs purified from CSF of AD-affected individuals. Furthermore, we show that modulation of BIN1 expression regulates the release of Tau via EVs *in vitro*. Altered BIN1 expression exacerbates Tau pathology and promotes Tau spreading *in vivo*. Finally, we report that the targeted deletion of *Bin1* from microglia resulted in a statistically-significant reduction in the expression of several heat-shock proteins, previously associated with Tau proteostasis, in microglial cells from male but not from female mice. Taken together, our observations suggest that BIN1 could contribute to the progression of AD-related Tau pathology by altering microglial Tau clearance and release in extracellular vesicles, thereby promoting the spread of Tau pathology throughout the brain.

## Results

### BIN1 and Tau are present in EVs purified from CSF

To gain insights into the mechanism of action of *BIN1*-associated genetic variants in AD etiology, we looked at their effect in relation to Aβ levels (measured by PET), as well as the amount of pTau181 (Threonine 181) in CSF (Methods)^22^. Both *BIN1* AD risk alleles rs6431223 (OR = 1.12, P = 2.5 × 10^−11^) and rs6733839 (OR = 1.22, P = 7 × 10^−44^)^22^ were significantly associated with increased levels of CSF pTau181 (*B* = 0.21, *P* = 0.02 and *B* = 0.17 *P* = 0.04), but were not correlated with amyloid deposition as monitored with PET imaging (Table 1).

**Table 1:** Effect of four SNPs associated with Alzheimer’s disease on amyloid levels (PET) and pTau 181 in CSF.

Gene	SNP id	AD Risk Allele	AD OR	Aβ *B*	Aβ *p-value*	p-Tau *B*	p-Tau *p-value*
BIN1	rs6733839	T	1.22	0.00	0.64	0.17	0.04
BIN1	rs6431223	G	1.12	0.00	0.97	0.21	0.02
APOE	rs429358	C	3.86	0.12	9.87E-35	0.11	0.02
ABCA7	rs4147929	A	1.15	0.04	3.61E-4	0.09	0.44

SNP = Single Nucleotide Polymorphism; AD = Alzheimer’s Disease; Aβ, Amyloid Beta levels as measured by positron emission tomography; p-Tau, phosphorylated Tau p181 levels measured in Cerebrospinal Fluid; AD Risk Allele = Allele associated with risk of developing Alzheimer’s Disease; OR = Odds Ratio; B, change in levels (effect size) in Standard Deviations (SDs) from the mean.

**Figure 1:**
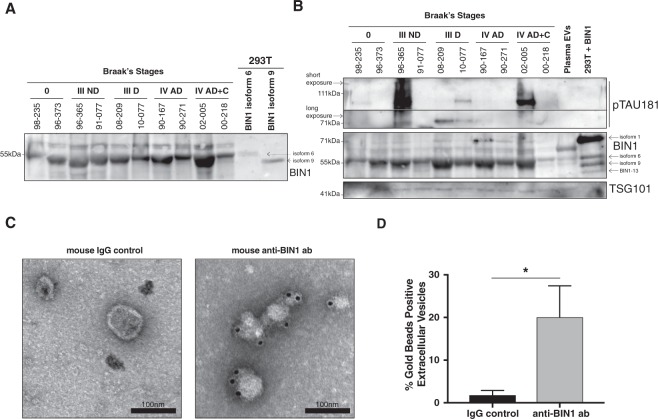
BIN1 and Tau are present in the EVs purified from CSF. (**A**,**B**) Western blotting of post-mortem CSFs samples (reference codes reported) obtained from individuals with neuropathological evaluation of Braak’s stages: 0: Non-Demeted Control, Braak’s Stage III Non-Demented (ND), Braak’s Stage III Demented, Braak’s Stage IV Alzheimer’s Disease, Braak’s Stage IV Alzheimer’s Disease with amyloid deposit C. (**A**) Western blotting of concentrated CSFs samples showed the presence of various isoforms of BIN1. As control, protein lysates from 293T cells transfected with plasmids expressing BIN1 isoform 6 or isoform 9 were loaded on the gel and indicated by arrows. True-Blot secondary antibodies (Rockland) had been used to avoid detection of non-specific bands. WB image shown represents cropped section of full blot shown in Supplementary Fig. 10. (**B**) Western blotting of EVs purified by SEC from CSF samples showed the presence of various isoforms of BIN1, pTau181 (short and long exposure indicated by arrows) and TSG101. As controls, EVs purified from Plasma of Healthy Donors and protein lysate from 293T cells co-transfected with plasmids expressing BIN1 isoform 1, 6, 9 and BIN1-13 were loaded on the gel, and indicated by arrows. Plasma EVs show the presence of BIN1, as previously reported^34^, as well as TSG101. True-Blot secondary antibodies (Rockland) had been used to avoid detection of non-specific bands. WB images shown represent cropped sections of full blots shown in Supplementary Fig. 10. (**C**) BIN1 and control IgG Immuno-EM was performed on EVs purified by SEC from pooled post-mortem CSF samples from individuals at Braak’s Stages III and IV. Magnification of one representative image out of 5 per sample. Scale: 100 nm. (**D**) Quantification of the percentage of BIN1^+^ and IgG^+^ EVs detected in fraction E1 + E2. Plot represents the mean and SD of three independent experiments performed. Paired parametric two-tailed Student’s t-test (95% confidence): p-value: *p < 0.05; **<0.01; ***p < 0.001.

Increased expression of BIN1 protein had been observed in plasma from AD-affected individuals^39^ and BIN1-containing extracellular vesicles have been detected in the human plasma^34^, raising the possibility that BIN1 might be associated with extracellular vesicles in other bodily fluids. Thus, we first investigated whether BIN1 could be found in post-mortem CSF samples from various individuals (Supplementary Dataset 2). We concentrated 100–200 μl of CSF from each sample and analyzed proteins by Western Blotting (WB). We detected various isoforms of BIN1 in all tested samples, regardless of Braak’s Stage (Fig. 1A).

It has been reported that the majority of pTau181 is present in exosomes purified from CSF of Mild AD affected individuals, and that BIN1 and Tau are present in exosomes purified from Tau-expressing cell line^40^. To determine whether BIN1 and Tau were colocalized in the same fraction containing exosomes purified from CSF, we implemented Sepharose Size-Exclusion Chromatography (SEC)^41^. Fraction 4, E1 and E2 purified from CSF by SEC, contained the exosome marker CD9, as well as Tau and different isoforms of BIN1 (Supplementary Fig. S1). Nanoparticle tracking analysis showed that fractions E1 and E2 contained extracellular vesicles ranging mainly between 80 and 300 nm (Supplementary Fig. S1). Given the varied definitions of exosome, we termed the vesicles in these fractions Extracellular Vesicles (EVs).

To address the potential role of BIN1 in AD-associated Tau pathology, we purified EVs from 20 samples of CSF from individuals with different Braak’s Stages and analyzed an initial 10 samples by WB demonstrating the presence of several BIN1 isoforms in all samples, as calibrated using lysates of HEK293T cells transfected with isoforms 1, 6, 9 and BIN1-13 (BIN1 isoform lacking exons 7, 13, 14, 15, 16 and 17, according to the new nomenclature) expressing plasmids (Fig. 1B). Upon analyzing our samples for Tau content, we detected phosphorylated and hyperphosphorylated (high molecular weight) pTau181 in 6/10 samples. Analysis of the second set of 10 samples, on a Tris-Acetate gel, demonstrated the presence of BIN1 in all the samples, as well as hyperphosphorylated pTau181 and oligomeric Tau (T22, previously validated^42^) in 8/10 samples (Supplementary Fig. S2). The presence of exosomes in EVs fractions was confirmed by the detection of exosome marker TSG101 (Fig. 1B and Supplementary Fig. S2). The limited set of samples precludes any conclusions about the relationship between CSF BIN1 levels and Braak’s Stage.

BIN1 has been found on the lipid membranes of microparticles from plasma^34^. To investigate whether BIN1 could be found on the lipid membranes of the EVs purified from CSF, we performed immuno-electron-microscopy (Immuno-EM) using an anti-BIN1 antibody in native conditions. We detected BIN1 on the lipid membrane of ≈20% of the EVs in fraction E1 + E2 (Fig. 1C,D).

### BIN1-associated Tau-containing EVs are seeding-competent

**Figure 2:**
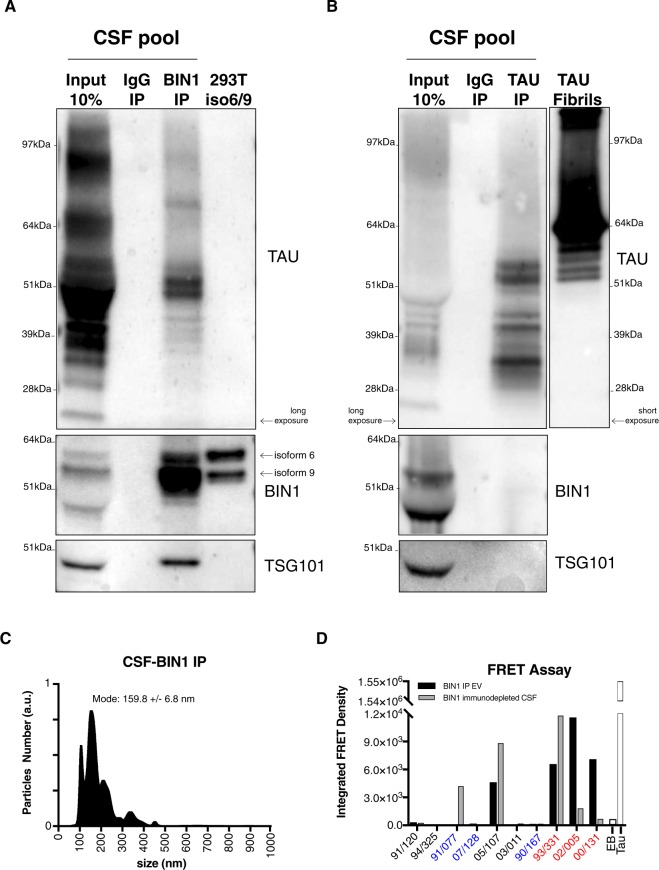
BIN1-associated, Tau-containing EVs are seeding-competent. (**A**) BIN1 immunoprecipitated EVs show the presence of various isoforms of Tau (long exposure shown) as well as BIN1 with molecular weights compatible with that of isoform 6 and 9 (as shown in lane containing protein lysate from 293T cells transfected with BIN1 isoform 6 and 9 expressing plasmids and indicated by arrows). TST101 represents exosome marker. True-Blot secondary antibodies (Rockland) had been used to avoid detection of non-specific bands. One representative experiment shown out of 3 independent replicates. WB images shown represent cropped sections of full blots shown in Supplementary Fig. 11. (**B**) Tau immunoprecipitated material shows the presence of various truncated and full-length Tau isoforms (long exposure shown) with molecular weights compatible with control aggregated Tau fibrils (short exposure shown). No BIN1 or TST101 was detected. True-Blot secondary antibodies (Rockland) had been used to avoid detection of non-specific bands. One representative experiment shown out of 3 independent replicates. WB images shown represent cropped sections of full blots shown in Supplementary Fig. 11. (**C**) Nanoparticle tracking analysis of extracellular vesicles purified by affinity immunopurification with anti-BIN1 from human CSF. One representative experiment out of 3 independent replicates. (**D**) BIN1 immunoprecipitated EVs (black bars) as well as BIN1 immunodepleted CSFs (gray bars) were prepared from samples obtained from 10 different individuals and tested in the Tau-FRET assay for Tau oligomerization. Results are plotted as Integrated FRET Density values for each sample (reference codes reported). Samples containing pTau and/or oligoTau by previous western blot analysis are highlighted in red. Samples devoid of pTau and/or oligoTau by previous western blot analysis are highlighted in blue. Elution Buffer (EB) serves as a negative control, whereas Tau fibrils (3 μM) serve as a positive control.

Given the localization of BIN1 on the lipid membranes of EVs, we asked whether we could immunopurify EVs from CSF with BIN1 antibody, using a methodology similar to that employed previously^34^. We pooled together CSF samples from 3–4 individuals (Braak’s Stages III-IV) for a total of 5 ml and performed BIN1, and Tau immunopurification (IP) as control for the presence of Tau in CSF. BIN1 antibody immunoprecipitated EVs contained varied monomeric and aggregated Tau isoforms (Fig. 2A). Conversely, Tau antibody immunoprecipitated mostly soluble truncated and monomeric Tau fibrils from CSF (Fig. 2B). Such observations support previous finding that located Tau in the inner leaflet of EVs^9^. Furthermore, material immunoprecipitated with BIN1 antibodies showed two distinct BIN1 isoforms, with a molecular weight compatible to the isoforms 6 and 9, as calibrated using lysates of HEK293T cells transfected with isoform 6 and 9 expressing plasmids (Fig. 2A). By contrast, material immunoprecipitated with Tau antibodies showed no BIN1 (Fig. 2B). The presence of exosomes in the BIN1 IP material, but not in Tau IP material, was confirmed by the detection of exosomes marker TSG101 (Fig. 2A,B). Nanoparticle tracking analysis revealed that BIN1 immunopurified EVs are comparable in size to the EVs purified by SEC (Fig. 2C and Supplementary Fig. S1). Taken together with the immuno-EM results, these data suggest that BIN1 is present on the surfaces of Tau-containing EVs.

Since we detected Tau species implicated in spreading Tau pathology in EVs from CSF, we investigated whether BIN1-associated EVs were seeding-competent, using the Tau-FRET assay^5^. HEK293T cells constitutively express the RD domain of Tau P301S fused with either CFP or YFP at C-terminal. Tau RD domain shows a diffuse cytoplasmic localization in these cells. Upon uptake of aggregation-competent Tau species, Tau RD domains bearing CFP (FRET donor) are brought into close proximity to YFP (FRET acceptor). The extent of Tau aggregation is then analyzed by flow cytometry. After BIN1 IP, EVs from 4/10 samples generated a positive FRET signal compared to controls (Fig. 2D, black bars). For several of the CSF samples analyzed in the FRET assay, EVs had been previously analyzed for the content of pTau and oligomerized Tau by WB (Fig. 1B and Supplementary Fig. S2). EVs that showed detectable pTau and/or oligomerized Tau by WB (93/331, 02/005 and 00/131; Fig. 1B and Supplementary Fig. S2), produced substantial FRET signal in comparison to controls (highlighted in red in Fig. 2D), indicating seeding competence, whereas EVs containing no or barely detectable pTau by WB (91/077, 07/128 and 90/167; Fig. 1B and Supplementary Fig. S2) generated no FRET signal (highlighted in blue in Fig. 2D). In parallel, we performed FRET assays using BIN1-immunodepleted CSF samples from the same individuals. FRET signals were detected in 3/10 samples, suggesting the presence of free-floating aggregation-prone Tau (Fig. 2D, gray bars). Taken together, our data suggested that BIN1 is associated with Tau-containing, seeding competent EVs in CSF samples from a substantial proportion of AD-affected individuals.

### Human BIN1 isoform 9 favors the release of Tau via EVs *in vitro* as well as increased area of pTau staining *in vivo*

**Figure 3:**
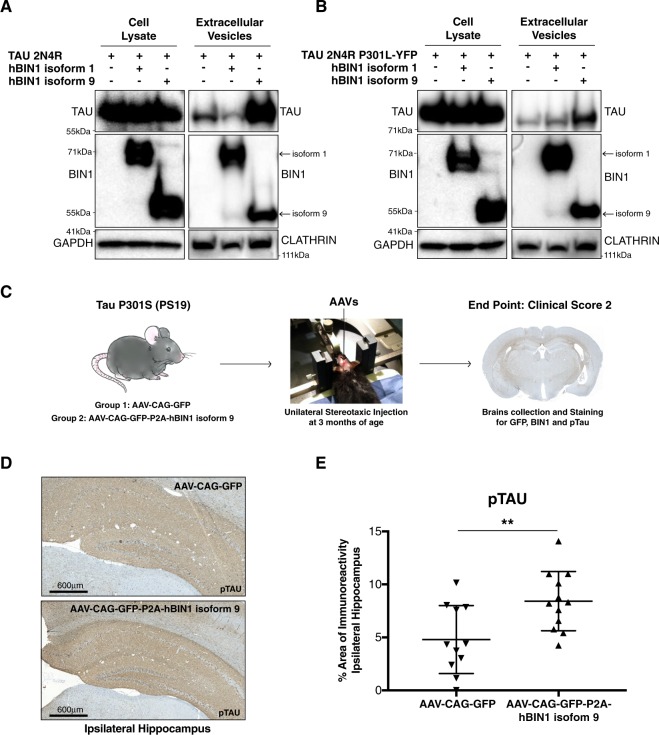
Human BIN1 isoform 9 favors the release of Tau via EVs *in vitro* as well as increasing the area of pTau staining *in vivo*. (**A**) Western blot of Tau and BIN1 in cell lysates (left panel) and EVs (right panel) from HEK293T cells transfected with Tau 2N4R alone or in presence of BIN1 isoform 1 or 9 (indicated by arrows). GAPDH (left panel) represents a cell lysate loading control. CLATHRIN (right panel) serves as an extracellular vesicle loading control. One representative experiment shown out of 4 independent replicates. WB images shown represent cropped sections of full blots shown in Supplementary Fig. 12. (**B**) Western blot of Tau and BIN1 in cell lysates (left panel) and EVs (right panel) from HEK293T cells transfected with Tau 2N4R P301L-YFP alone or in presence of BIN1 isoform 1 or 9 (indicated by arrows). GAPDH (left panel): cell lysate loading control. CLATHRIN (right panel): extracellular vesicle loading control. One representative experiment shown out of 4 independent replicates. WB images shown represent cropped sections of full blots shown in Supplementary Fig. 12. (**C**) Experimental scheme. Artwork by Channa Bao. (**D**) Representative image of Immunohistochemical staining of pTau (40E8) in hippocampi of PS19 mice injected with AAV expressing GFP or GFP-P2A-hBIN1 isoform 9. Scale: 600 μm. (**E**) Plot representing the percentage of pTau staining area in the ipsilateral hippocampus (area of pTau staining normalized to the size of the ipsilateral hippocampus for each animal). Each triangle represents one animal. Unpaired parametric Student’s t-test two tailed, 95% confidence: p-value: *p < 0.05; **<0.01; ***p < 0.001.

Since BIN1 isoform 9 is ubiquitously expressed^27^, and it is also the most abundantly detected isoform in Tau-containing, seeding-competent EVs from CSF (Fig. 2A), we sought to investigate whether over-expression of BIN1 would affect Tau release via EVs *in vitro*. Given ubiquitous expression of BIN1 isoform 9, we chose a non-CNS-related cell line (HEK293T) as model to test this hypothesis. HEK293T cells do not express Tau and spontaneously release extracellular vesicles^43^. We transfected 293T cells with plasmids expressing Tau 2N4R or Tau 2N4R P301L-YFP, in presence or absence of a plasmid expressing BIN1 isoform 9, or BIN1 isoform 1 (to control for isoform specificity). Forty-eight hours after transfection, we collected conditioned media (CM) and prepared EVs from it by differential ultracentrifugation, and generated protein lysates from the cells. Of note, we showed a similar size distribution of the EVs purified by all three methods: differential ultracentrifugation, size-exclusion chromatography and BIN1 IP (Fig. 2C, Supplementary Fig. S1). While BIN1 isoforms 9 and 1 expression did not affect overall Tau expression in 293T cells (Fig. 3A, left panel), an increased amount of Tau was found in EVs purified from CM collected from cells co-transfected with BIN1 isoform 9, in comparison to EVs from cells transfected with Tau alone (Fig. 3A right panel, Supplementary Fig. S3). Experiments performed using a plasmid expressing Tau 2N4R P301L-YFP confirmed the effect of BIN1 isoform 9 in promoting the release of Tau via EVs, regardless the presence of mutant Tau (Fig. 3B, Supplementary Fig. S3). The expression of BIN1 isoform 1 did not affect the amount of Tau found in EVs purified from CM when compared with the content of Tau detected in EVs purified from CM of cells transfected with Tau-expressing plasmid alone. Expression of BIN1 isoform 9 results in an increased amount of Tau found in EVs purified from CM above the basal level of Tau found in EVs when cells are transfected with plasmids expressing exclusively Tau or Tau and Bin1 isoform 1, suggesting an isoform-specific role for BIN1 in promoting Tau-containing EVs release (Fig. 3A,B).

Since BIN1 isoform 9 was increased in post-mortem brain samples from AD affected individuals and its amount positively correlated with NFTs in the same samples^37^, we sought to determine if over-expression of BIN1 isoform 9 exacerbates Tau pathology *in vivo*. Therefore, we stereotactically injected AAVs expressing GFP-P2A-hBIN1 isoform 9 or AAV-GFP control, into the hippocampus of Tau P301S-expressing mice (PS19)^44^. Since BIN1 isoform 9 can be detected ubiquitously in the brain^29,30,45^, we chose a cell-type agnostic approach and used a mix of serotypes 1 and 8 to infect a broad spectrum of cells^46^, as well as constitutive non-cell specific CAG promoter. Mice were injected at 3 months of age, before the onset of Tau pathology, and sacrificed upon reaching clinical score 2 (Methods), when brains were harvested, fixed, sectioned and stained for GFP, BIN1 and pTau (phospho-Ser 202/phospho-Thr 205, clone 40E8, previously validated^47^) (Fig. 3C). No statistically significant difference was observed in the time required to reach clinical score 2 between the two groups, ranging between 250 and 300 days. As determined by GFP and NeuN, GFAP or Iba1 co-staining, we transduced neurons, astrocytes, and a low number of microglia cells (Supplementary Fig. S4). AAV(1N8)-CAG-GFP control and CAG-GFP-P2A-hBIN1 isoform 9 infection resulted in similar distribution of GFP immunoreactivity (Supplementary Fig. S5), while AAV(1N8)-CAG-GFP-P2A-hBIN1 isoform 9 drove substantial BIN 1 expression (Supplementary Figs S4 and 5). Interestingly, a statistically significant increase in the percent area of pTau immunoreactivity was demonstrated in the ipsilateral hippocampus of mice overexpressing BIN1 isoform 9 in comparison to mice injected with AAV(1N8)-CAG-GFP control (Fig. 3D,E) consistent with the hypothesis that BIN1 is implicated in Tau pathology.

Subsequently, we investigated whether over-expression of BIN1 isoform 1 would produce a similar result. Given the expression of BIN1 isoform 1 exclusively in neurons^33,48^, we chose the neuronal-specific *SYNAPSIN 1* promoter to drive the expression of BIN1 isoform 1. PS19 mice were injected at 3 months of age and sacrificed following a similar timeline to that previously used. As determined by GFP and NeuN, GFAP or Iba1 co-staining, we had been able to obtain a productive infection exclusively in neurons (Supplementary Fig. S6), and no statistically significant difference in the GFP staining between viruses suggests that they had similar infectivity (Supplementary Fig. S7). While AAV(1N8)-hSYN-GFP-P2A-hBIN1 isoform 1 drove substantial BIN 1 expression (Supplementary Figs S6 and S7), no difference in the percent area of pTau immunoreactivity in the hippocampus was observed between mice overexpressing BIN1 isoform 1 and mice injected with AAV(1N8)-hSYN-GFP control (Supplementary Fig. S7). This observation, along with the failure of BIN1 isoform 1 to promote Tau release in EVs *in vitro*, suggests a structural specificity of ubiquitous BIN1 isoform 9 in the context of Tau pathology.

### Identification and quantification of BIN1 isoform expression in human and mice

**Figure 4:**
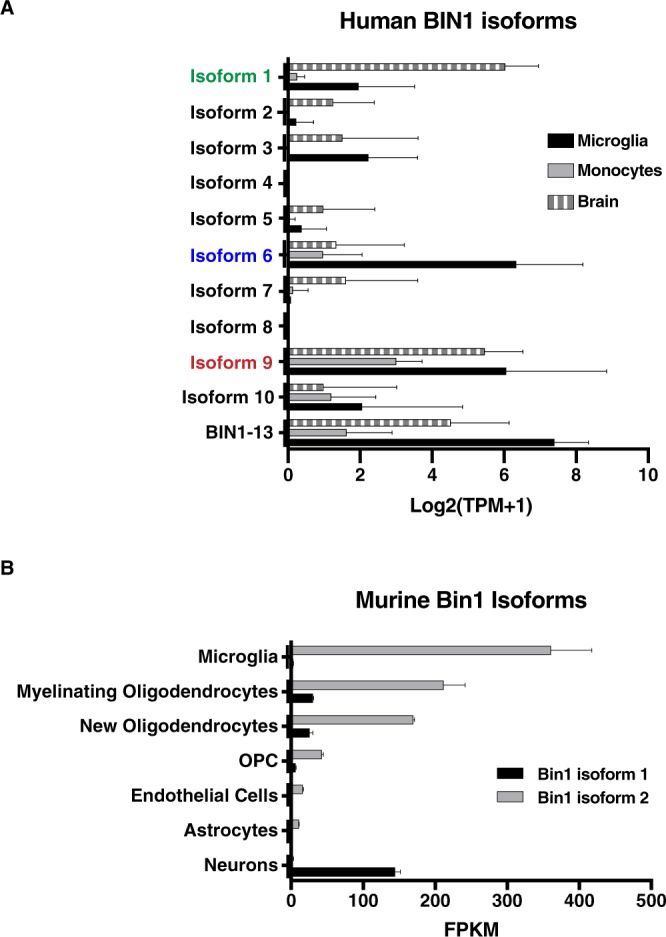
Identification and quantification of BIN1 isoform expression in human and mice. (**A**) Plot representing human *BIN1* isoforms transcript expression in total brain lysate (striped bars), in microglia (black bars) and in monocytes (gray bars). Isoform 1 indicated in green; isoform 6 indicated in blue; isoform 9 indicated in red. Data are expressed in Log2 Transcript per million +1 (Log2 (TPM + 1)). (**B**) Plot representing murine *Bin1* isoform 1 (black bars) and isoform 2 (gray bars) transcript expression in various CNS cell types. Data are expressed in Fragments Per Kilobase Million (FPKM).

In human brain, oligodendrocytes and microglia cells express the highest levels of *BIN1* transcripts^45^. To evaluate microglial *BIN1* isoform expression, we reprocessed sequencing data coming from direct *ex vivo* RNAseq analysis of human microglia as compared to human brain lysate and monocytes^49^. *BIN1* isoforms 1, 9 and BIN1-13 are the most abundant in brain lysate (Fig. 4A, striped bars), while isoforms 6, 9 and BIN1-13 are the major *BIN1* transcripts expressed by microglia (Fig. 4A, black bars). Interestingly, human *BIN1* isoform 6 and BIN1-13 may be relatively specific for microglia among mononuclear phagocytes, as monocytes express them at considerably lower levels (Fig. 4A, gray bars).

In mice, according to ensemble database, *Bin1* gene is expressed in 2 transcript variants: *Bin1* transcript ENSMUST00000025239 encodes for *Bin1* isoform 1 (Uniprot O08539-1) and shows 94.8% homology with human *BIN1* isoform 1. *Bin1* transcript ENSMUST00000091967 encodes for *Bin1* isoform 2 (Uniprot Q6P1B9) and shows 95% homology with human *BIN1* isoform 6. We reprocessed RNAseq data from adult mouse brain^50^ and associated the expression level of the different isoforms with specific brain cell types. As observed in analysis of human cells, *Bin1* isoform 1 is mostly expressed in neurons (Fig. 4B, black bars), while *Bin1* isoform 2 is expressed by glial cells. Interestingly, the cell types that express *Bin1* isoform 2 at the highest level in murine brain are microglia and myelinating oligodendrocytes (Fig. 4B, gray bars). Murine *Bin1* isoform 2 is relatively specific for microglia among myeloid cells, as it is expressed at negligible levels by monocytes, peritoneal macrophages and bone marrow-derived macrophages^51^. Of note, the murine homologous of human *BIN1* isoform 9 (Uniprot O08539-2) had been cloned from a mouse embryonal tissue cDNA library^52^ and its expression has been detected in adult mouse brain^53^.

To summarize, human microglia express high amounts of the transcript expressing ubiquitous *BIN1* isoforms 9, as well as the transcripts of microglia-specific isoforms 6 and BIN1-13, while mouse microglia express the transcript encoding *Bin1* isoform 2, homologous to human *BIN1* isoform 6.

### Deletion of murine microglial Bin1 reduces the release of Tau via EVs *in vitro* and decreases pTau spreading *in vivo*

Given the homology between human BIN1 isoform 6 and murine Bin1 isoform 2, we first tested whether over-expression of murine transcript variant 2 would affect Tau release via EVs in 293T cells. Cells co-transfected with plasmids expressing Tau P301S and murine Bin1 isoform 2 showed a statistically significant increased release of Tau via EVs in comparison to cells exclusively expressing Tau P301S (Supplementary Fig. 8).

**Figure 5:**
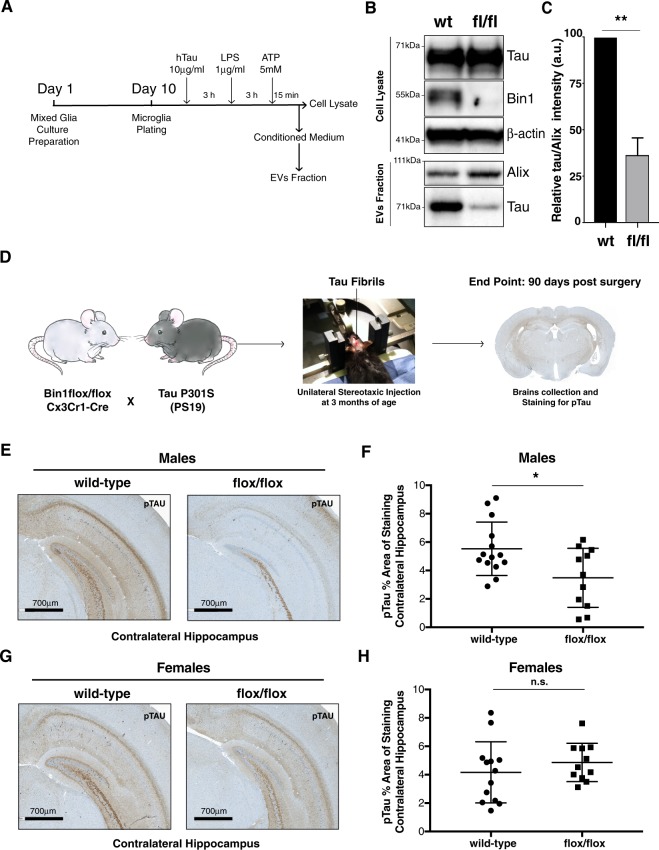
Deletion of Bin1 in microglia reduces the release of Tau via EVs *in vitro* as well as decreases pTau spreading in male mice *in vivo*. (**A**) Experimental timeline for *in vitro* microglia-derived Tau-containing EVs analysis. (**B**) Western blot of Tau and BIN1 in cell lysates (top) and EVs (bottom) from Bin1 wt and cKO primary microglia cells after administration of Tau fibrils and stimulation with LPS and ATP, as reported in A. β-actin: cell lysate loading control. Exosome marker Alix: EVs loading control. Each experiment performed by pooling together microglia from more than 10 pups of the same genotype. One representative experiment shown out of 4 independent replicates. WB images shown represent cropped sections of full blots shown in Supplementary Fig. 13. (**C**) Plot representing densitometric analysis of Tau band intensity normalized to Alix band intensity, as obtained by WB from EVs purified from CMs of microglia Bin1 wt and cKO, respectively. Plot represents mean and SD of 4 independent replicates. Paired parametric Student’s t-test two tailed, 95% confidence: p-value: *p < 0.05; **<0.01; ***p < 0.001. (D) Experimental scheme. Artwork by Channa Bao. (**E**) Representative images of immunohistochemical staining of pTau (40E8) in contralateral hippocampi of *Bin1*^*flox/flox*^*:: Cx3Cr1-Cre*^+^*::PS19*^*Hemi*^ and *Bin1*^*wt*^*:: Cx3Cr1-Cre*^*−*^*::PS19*^*Hemi*^ littermate male mice injected with Tau fibrils. Scale: 700 μm. (**F**) Plot representing the percentage of pTau staining area normalized to the size of the hippocampus for each animal. Each symbol represents one animal. (**G**) Representative images of immunohistochemical staining of pTau (40E8) in contralateral hippocampi of *Bin1*^*flox/flox*^*:: Cx3Cr1-Cre*^+^*:: PS19*^*Hemi*^ *and Bin1*^*wt*^*:: Cx3Cr1-Cre*^*−*^*:: PS19*^*Hemi*^ littermate female mice injected with Tau fibrils. Scale: 700 μm. (**H**) Plot representing the percentage of pTau staining area normalized to over the size of the hippocampus for each animal. Each symbol represents one single animal. Two-way ANOVA with Holm’s p-value adjustment (two-sided) (see Supplementary Fig. 9): p-value: *p < 0.05; **<0.01; ***p < 0.001.

Since we have identified that BIN1 isoform 6 is relatively specific for microglia in human, the presence of human BIN1 isoform 6 in CSF-derived EVs suggests a microglial origin for these EVs. In a murine model of Tau pathology, microglia depletion or inhibition of exosome synthesis reduced Tau spreading^9^. Therefore, we hypothesized that BIN1 in microglia may promote Tau spreading. Microglia don’t express *MAPT* gene and internalize Tau by endocytosis/phagocytosis^54^. To initiate our studies of the effect of the loss of *Bin1* in microglia for spreading of Tau pathology, we analyzed cell-lysates and CMs for Tau, using primary murine microglia from cKO and WT littermate mice, after incubation with human Tau 2N4R fibrils (Fig. 5A) and stimulation with TLR4 ligand LPS to induce Tau ubiquitination (a cue for insertion in multi-vesicular bodies), and purinergic receptors ligand ATP to induce exocytosis of exosomes^9^. The size distribution of EVs purified by commercial SEC columns from microglial CM was comparable to that found using the previously-applied methodologies (Supplementary Fig. 1). We observed a statistically significant reduction of Tau in the EVs purified from microglial *Bin1*-cKO CM as compared to CM from microglia of *WT* littermates (Fig. 5B,C). Of note, analysis of cell lysates revealed the presence of 2 anti-BIN1 antibody immune-reactive bands, with a molecular weights compatible to murine Bin1 transcript variant 2 ((Uniprot Q6P1B9) homologous to Human BIN1 isoform 6), as well as the murine homologous of human *BIN1* isoform 9 (Uniprot O08539-2), confirming previous observations^53^. Importantly, cell lysates analysis excluded an effect of *Bin1* deletion on Tau uptake, suggesting that loss of microglial BIN1 directly reduces incorporation of Tau in EVs.

To determine whether microglial deficiency for *Bin1* would affect Tau pathology *in vivo*, we crossed *Bin1*-cKO with PS19 mice. At 3 months of age, mice were stereotactically injected in the hippocampus with Tau fibrils^55^, and brains were harvested 90 days after surgery, sectioned and stained for pTau (Fig. 5D). Unexpectedly, male but not female homozygous microglial-*Bin1*-deficient mice showed a statistically significant reduction of Tau spreading to the contralateral hippocampus in comparison to *Bin1*-expressing littermates (Fig. 5E–H). Mice with loss of one microglial *Bin1* allele showed no statistically significant effect on the extent of Tau pathology, regardless of sex (Supplementary Fig. 9).

### Loss of Bin1 in microglia affects gene expression in a sexually dimorphic fashion

**Figure 6:**
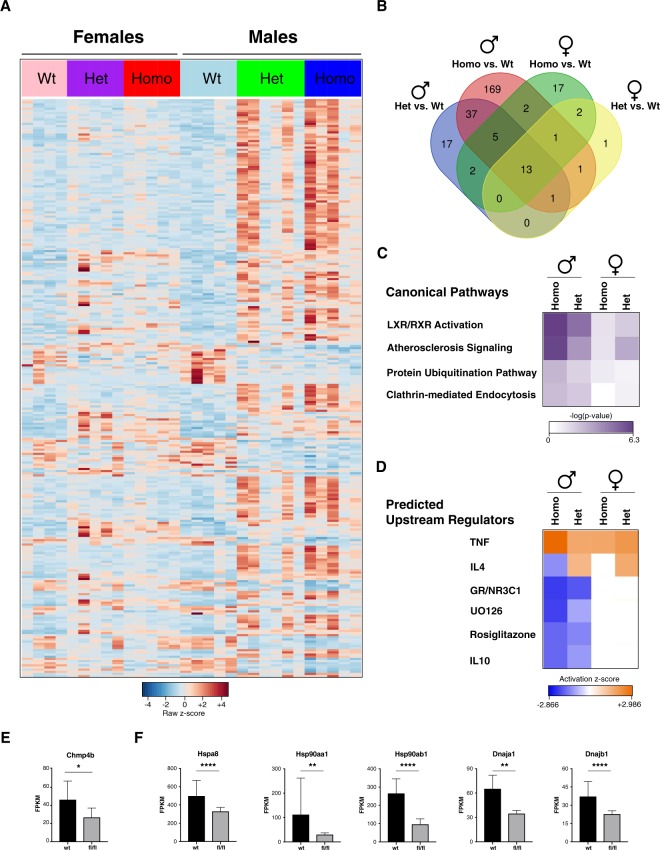
Deletion of Bin1 in microglia affects gene expression in a sexually dimorphic fashion. (**A**) Heatmap representing FPKM values of 268 differentially expressed genes in microglia from *Bin1*^*flox/flox*^*::Cx3Cr1-Cre*^+^, *Bin1*^*wt/flox*^*::Cx3Cr1-Cre*^+^ and *Bin1*^*wt/flox*^*::Cx3Cr1-Cre*^*−*^ male and female mice (Average Normalized Counts > 4; Fold Change |FC| 1.5; padj < 0.05). (**B**) Venn diagram representing the distribution of 268 differentially expressed genes across genotypes and sexes. (**C**) Selection of the most statistically significant Canonical Pathways obtained by Ingenuity Pathway Analysis of the DEGs comparison of *Bin1*^*flox/flox*^*::Cx3Cr1-Cre*^+^(Homo KO) versus *Bin1*^*wt/flox*^*::Cx3Cr1-Cre*^*−*^ (WT) microglia from male mice (named Homo) and the DEGs comparison of *Bin1*^*wt/flox*^*::Cx3Cr1-Cre*^+^(Het KO) versus *Bin1*^*wt/flox*^*::Cx3Cr1-Cre*^*−*^ (WT) microglia from male mice (named Het). (**D**) Selection of the most statistically significant Predicted Upstream Regulators obtained by Ingenuity Pathway Analysis of the DEGs comparison of *Bin1*^*flox/flox*^*::Cx3Cr1-Cre*^+^(Homo KO) versus *Bin1*^*wt/flox*^*::Cx3Cr1-Cre*^*−*^ (WT) microglia from male mice (named Homo) and the DEGs comparison of *Bin1*^*wt/flox*^*::Cx3Cr1-Cre*^+^(Het KO) versus *Bin1*^*wt/flox*^::*Cx3Cr1-Cre*^*−*^ (WT) microglia from male mice (named Het). (**E**,**F**) Plots representing mRNA expression (mean ± SD) of *Cmp4b*, *Hspa8*, *Hsp90aa1*, *Hsp90ab1*, *Dnaja1* and *Dnajb1* in WT and *Bin1*-cKO microglia from male mice, expressed as Fragments Per Kilobase Million (FPKM). Adjusted p-value: *padj < 0.05; **padj < 0.01; ***padj < 0.001; ****padj < 0.0001.

Sexual dimorphism had been previously observed in mosaic *Bin1* knock-out mice, in response to the induction of experimental colitis^56^. Given that microglial *Bin1*-cKO mice showed sexual dimorphism with regard to Tau spreading, we determined the effect of the loss of *Bin1* on microglial gene expression in male and female mice. Transcriptomes of FACS-sorted microglia from 3 month-old *Bin1*^*flox/flox*^*::Cx3Cr1-Cre*^+^ mice and WT littermates were analyzed using RNAseq. We observed a highly significant number of differentially expressed genes (DEGs) in microglia lacking *Bin1* in male as compared with female mice (Fig. 6A). In particular, microglia from *Bin1*^*flox/flox*^*::Cx3Cr1-Cre*^+^ male mice showed 229 statistically significant DEGs in comparison to WT males (Fig. 6B), while microglia from the corresponding female mice showed just 19 DEGs when compared to WT females (Fig. 6B). Upon comparison of microglia from males and females by Ingenuity Pathway Analysis, the top over-represented canonical pathway was LXR/RXR activation (Fig. 6C), the top predicted upstream activator was TNF, and top predicted upstream inhibitor was Glucocorticoid Receptor (Fig. 6D). While the loss of *Bin1* in microglia resulted in an increase of *Cst7*, *Chst1*, *Chst2*, *Pdcd1*, *Lox*, *Rxrγ*, *Gpr153*, *Sp100* and *Hcar2* transcripts regardless of sex, the major difference between males and females appeared in the list of the repressed genes (Supplementary Dataset 2). Remarkably, comparison of gene expression in microglia from female *Bin1*^*flox/flox*^*::Cx3-Cr1-Cre*^+^ and *Bin1*^*flox/wt*^*::Cx3-Cr1-Cre*^+^ mice to WT littermates showed respectively 0 and 1 mRNAs statistically significantly repressed. By contrast, microglia from male mice heterozygous or homozygous cKO for *Bin1* showed repression of approximately 1/3 of DEGs compared to WT littermates. Interestingly, loss of 2 copies of *Bin1* resulted in *Charged Multivesicular Body Protein 4B* (*Chmp4b*) downregulation in microglia (Fig. 6E). CHMP4B/BIN1 interaction has previously been linked to release of BIN1-containing microparticles by cardiomyocytes^34^. Furthermore, microglia from *Bin1*^*flox/flox*^*::Cx3Cr1-Cre*^+^ male mice showed a statistically significant repression of genes, encoding chaperone proteins, such as *Hspa8* (Hsp70), *Hsp90aa1*, *Hsp90ab1*, *Dnaja1* and *Dnajb1* (Fig. 6F). Consistent with a wider role for BIN1 in Tau pathology, these genes had been previously linked to Tau proteostasis and clearance^57^.

## Discussion

In this study, we show that BIN1 and Tau are present in seeding-competent EVs purified from CSF of AD-affected individuals. Additionally, we report that the over-expression of the ubiquitous BIN1 isoform 9, but not neuronal isoform 1, favors Tau release via EVs *in vitro* and exacerbates Tau pathology *in vivo*. Consistent with a role for BIN1 in EV-mediated Tau spreading, we found that loss of microglial Bin1 reduced the release of Tau via EVs *in vitro* and decreased Tau spreading *in vivo*. Unexpectedly, this observation was made exclusively in male mice. Finally, we reported that deletion of Bin1 resulted in a statistically significant reduction in the expression of several heat-shock proteins in microglia from male but not from female mice.

Variation at the *BIN1* locus has shown a consistent statistically-significant association to LOAD^22,23^, although the mechanistic link between BIN1 and AD has remained obscure. In the present study, we focused on the relationship between BIN1 and Tau spreading, based on the correlation between AD-associated *BIN1* SNPs and expression levels with Tau pathology^36,37^. Our analysis confirmed and extended the observation that *BIN1* SNPs are associated with total Tau and pTau, by showing that SNPs rs6431223 and rs6733839 positively correlate with increased levels of pTau181 in the CSF. Also, we confirmed the absence of correlation between the analyzed *BIN1* SNPs and the amount of amyloid-PET tracer retention. Subsequently, we showed that BIN1-Tau association can be detected *ex vivo* in EVs purified from human CSF. While such results had been limited to the use of post-mortem samples, our findings are consistent with previous reports showing that the majority of AD-CSF pTau181 is present in exosomes and that BIN1 and Tau are present in exosomes purified from Tau-expressing cell lines^40^. Such observations suggest the involvement of BIN1 in endosomal trafficking as well as exosome secretion. We also showed that BIN1 and pTau/oligomeric Tau-containing EVs from CSF of AD-affected individuals are seeding-competent *in vitro*, linking BIN1 with Tau spreading. In this regard, our data confirm and extend previous reports showing that extracellular vesicles purified from either clinical CSF samples or various murine Tau pathology models contain seeding-competent Tau as defined in the Tau-FRET aggregation assay^10–12,58^.

The immune-purification of EVs from human CSF revealed the presence of BIN1 isoforms 6 and 9. It has been reported that the level of ubiquitously expressed BIN1 isoform 9 protein was increased in AD post-mortem brain samples regardless of *BIN1* genotype and that BIN1 levels positively correlated with NFTs in the same samples^37,38^. Here, we show that expression of BIN1 isoform 9 favors the release of Tau via EVs *in vitro*. Furthermore, we observed that AAV-mediated over-expression of the ubiquitous BIN1 isoform 9 in neurons, astrocytes and microglia, increases the percent area of hippocampal pTau immunoreactivity *in vivo* in comparison to ubiquitous expression of AAV control in a model of Tau pathology. While we acknowledge that multiple mechanisms could contribute to such *in vivo* observation, this result still suggests that the expression of ubiquitous BIN1 isoform 9 could exacerbate Tau pathology in the brain potentially via Tau-containing EVs. To our knowledge, this experiment is the first showing that modulation of BIN1 expression *in vivo* affects Tau pathology.

Interestingly, we observed that the AAV-mediated over-expression of the neuronal BIN1 isoform 1 specifically in neurons doesn’t affect the percent area of hippocampal pTau immunoreactivity *in vivo* in comparison to neuronal expression of AAV control in a model of Tau pathology. The major difference between BIN1 isoform 1 and 9 is that the former retains exon 7 and the CLAP (Clathrin and AP2) binding domain, previously linked to endocytosis and endosomes formation^31,59^. Thus, it is plausible that BIN1 isoform 1 and 9 might differentially regulate Tau endosomal trafficking due to their structural differences. Furthermore, since SH3 domain of BIN1 had been previously indicated as the binding partner for Tau^60^, it is possible that the CLAP domain in BIN1 isoform 1 might interfere with its SH3 domain in binding to Tau. Recently, it has been reported that the loss of neuronal BIN1 promotes Tau spreading in an *in vitro* microfluidic model^7^. It will be useful to reconcile this observation with our negative findings in an *in vivo* model.

Given the location of the AD-associated SNPs in the intergenic region upstream of the *BIN1* transcription start site, it’s plausible that its expression levels are affected by the risk allele^36,61^. However, direct evidence in favor of this possibility is scarce, likely because human *BIN1* is expressed as multiple isoforms in differing amounts by various brain cell types. Further, disease-mediated changes in the relative numbers of the various *BIN1*-expressing CNS cells could affect the results obtained by bulk RNAseq expression analysis of post-mortem brain samples. Interestingly, non–neuronal BIN1 isoforms are highly expressed in the brain particularly in microglia^49,51^. In mice, the *Bin1* locus contains a microglia-specific super-enhancer^51^. In humans, it has been shown that *BIN1* expression is significantly higher in microglia in comparison to monocytes^49^. Interestingly, *BIN1* SNP rs6733839, which we found positively correlated with pTau181 in CSF, lies in proximity to a PU.1 peak in human microglia^49^. We inspected this region and found that the change from common allele C to risk allele T generates a MEF2 consensus-binding site (TTAAAAA**C**AC > TTAAAAA**T**A). Interestingly, it has been shown that MEF motifs are enriched around microglia-specific PU.1 peaks in mice and in humans^49,51^, suggesting that MEF2 proteins could act as co-regulators of PU.1 transcription factor activity in microglia. Among the four genes that encode for MEF proteins (A-D), MEF2C is highly expressed in human microglia^49^, and the *MEF2C* locus contains a GWAS risk variant for LOAD^22^. Taken together these observations suggest that AD-associated *BIN1* SNP rs6733839 might affect *BIN1* expression in a cell-specific manner and points toward microglia as the relevant cell type.

It has been previously shown that microglia might favor Tau spreading in the brain^9,62^ and it has been proposed that exosomes of microglial origin could contribute to this process^9^. Our analysis showed that human microglia express high levels of the ubiquitous BIN1 isoform 9 and that isoform 9 is detectable in EVs purified from human CSF. Additionally, we linked isoform 9 to EV-mediated Tau release *in vitro* as well as exacerbation of Tau pathology *in vivo*. Of note, a limitation of our study is that microglia could not be selectively transduced due to lack of appropriate AAV vectors. The observation that over-expression of ubiquitous BIN1 isoform 9 via AAV by multiple cell types could exacerbate Tau pathology suggests that isoform 9 may be the primary BIN1 isoform responsible for EVs-mediated Tau spreading and that multiple cell types in the brain could be responsible for Tau-containing EVs secretion. Nevertheless, we found that human microglia express also BIN1 isoform 6, which we likewise detected in EVs purified from human CSF. Human BIN1 isoform 6 is homologous to murine *Bin1* isoform 2, which is primarily expressed by microglia together with putative murine homologous of human BIN1 isoform 9. In our studies, we showed that over-expression of Bin1 isoform 2 results in an increased release of Tau via EVs *in vitro* to an extent comparable to human BIN1 isoform 9. Furthermore, we observed that *ex-vivo* microglia lacking *Bin1* showed a decreased release of Tau via EVs, and we observed that male mice lacking microglial *Bin1* showed decreased Tau spreading *in vivo* in a murine model of Tau pathology. Taken together, such observations suggest that microglia could be major cell type implicated in the BIN1-involving EVs-mediated Tau spreading. Consistent with our overall hypothesis regarding BIN1, deletion of *Amph* gene – the *Drosophila* BIN1 homolog that lacks the CLAP domain and is not involved in endocytosis – reduces Tau toxicity in *Drosophila*^36^.

To understand the role of endogenous BIN1 in microglia, we performed RNAseq on microglia from *Bin1* cKO mice. To our surprise, we observed that loss of Bin1 in microglia deeply affected gene expression in male mice, but to a lesser extent in female mice, consistent with our observation that male, but not female, *Bin1*-cKO mice showed decreased Tau spreading *in vivo*. In microglia from males, the loss of *Bin1* results in downregulation of genes, encoding chaperone proteins, such as *Hsc7*0, *Hsp90aa1*, *Hsp90ab1*, *Dnaja1* and *Dnajb1*. Hsc70 expression is increased in AD model mice and patient brains^63^, and its inhibition promotes Tau degradation^64^. Furthermore, it has been shown recently that Hsc70/Dnaj chaperone complexes promote the extracellular release of Tau^65^. Hsp90 co-immunoprecipitates with Tau, and its inhibition reduces levels of pTau in cell cultures as well as in AD mouse models^66,67^. *Dnaja1* is the primary co-chaperone of the constitutively expressed HSC70 and antagonizes its stabilizing activity towards Tau^68^. In a *Drosophila* tauopathy model, *DnaJb1* knockdown stabilized cellular Tau levels^69^. Such observations support the hypothesis that BIN1 could affect Tau clearance in microglia by altering the expression of chaperone proteins previously associated with Tau proteostasis.

We also observed a down-regulation of the ESCRT-III pathway component *Chmp4b* in *Bin1-*cKo microglia. It has been reported that BIN1 directly interacts with CHMP4B, suggesting that BIN1 may act as an early ESCRT factor^34^. Thus, alteration of this pathway could result in a reduction of exosome generation and release, and this in turn could explain decreased Tau spreading as observed *in vivo*.

Taken together, our results suggest a model where AD-associated BIN1 SNPs initially promote increased BIN1 expression in microglia. Downstream of this enhanced BIN1 expression, we propose altered levels of chaperone proteins, which would modulate Tau clearance. Additionally, increased BIN1 expression could promote Tau secretion via extracellular vesicles, as a means of waste disposal. Together, these events would result in an increased spreading of pathogenic Tau and progression of AD.

To our knowledge the present report is the first to shed light on the role of BIN1 *in vivo* in the context of AD. Nevertheless, the strength of correlation between BIN1-associated SNPs and LOAD, the multiplicity of isoforms in which BIN1 is expressed, as well as the combination of the expression of different isoforms in different brain cell types involved in AD pathogenesis support the likelihood of BIN1 contributing to AD pathogenesis through multiple parallel mechanisms acting in different CNS cells. For these reasons, our study of BIN1’s role in AD represents a starting point in the effort to use genetic variation to gain a better understanding of overall disease pathogenesis.

## Material and Methods

### Ethical Statement

All experimental procedures involving animals were approved by the Institutional Animal Care and Use Committee (IACUC) at Biogen, and every effort was made to minimize suffering. All experiments involving animals were performed in accordance with relevant guidelines and regulations. Studies of human CSF samples were performed without individually identifiable information. Research undertaken on such specimens does not meet the regulatory definition of human subjects’ research.

### Human samples collection

Samples from post-mortem Cerebrospinal Fluids were obtained from the Netherlands Brain Bank (http://www.brainbank.nl) (Supplementary Dataset 2). Informed consent was obtained from the donor or for the donor from the next of kin for the use of the material and clinical data for research purposes. Control subjects were matched to AD subjects for sex, age, and post-mortem delay (pmd). All diagnoses were based on clinical assessment and neuropathological evaluation by experienced neuropathologists according to Braak’s classification and Amyloid deposition^1,2^. All CSF samples were spun at 300 g for 10 min to remove floating cells prior to freezing and storage at −80 °C.

### Genetic analysis

Twenty-two genetic variants associated with Alzheimer’s Disease^22^ were selected to evaluate their potential effect on amyloid levels (as measured by PET AV45 scans) or for their effect on CSF pTau181. The specific methodology employed here has been described elsewhere^70,71^.

### Extracellular Vesicle purification

Prior to Extracellular Vesicle (EVs) purification, samples were spun at 2000 g for 10 min at 4 °C and subsequently at 10,000 g for 30 min at 4 °C.

Custom Sepaharose Size-Exclusion Chromatography (SEC) has been used to purify EVs from CSF post-mortem samples as reported previously^41^. Briefly, Sepharose CL-2B (Cat:17-0140-01, GE healthcare) was stacked and packed to 15 ml in a 20 ml Biorad Econo-pac chromatography column (Cat:732-1010, Biorad). After equilibrating the matrix with 3 times the volume of PBS, 5 ml of sample was loaded on to the column. Elution was performed by gravity with 13 ml of PBS. The first 5 ml was collected as void, and then fractions 1, 2, 3, and 4 (1.5 ml each) were collected sequentially, followed by fractions E1 and E2 (1 ml each). Fraction 4, E1, and E2 were concentrated using Millipore Amicon 10KDa 2 ml/0.5 ml centrifugal filters. Samples are first concentrated by spinning at 7,500 g for 30 min using a 2 ml column (Cat: UFC201024), which yielded approximately 120 μl of sample. Then, PBS was added to the samples to a total volume of 500 μl, and a final concentration was performed by loading the samples on a 0.5 ml column (Cat: UFC501024, EMD Millipore) and spinning at 14,000 g for 30 min. These concentrated samples were used for downstream analysis.

Biotinylated antibody-based immunoaffinity purification was used to purify EVs from post-mortem CSF according to the manufacturer’s instructions (CSFLOWBASICA-1, System Bioscience). For Bin1 antibody sc-13575, biotinylation was performed using EZ-Lin TM NHS-PEG Solid Phase Biotinylation Kit spin columns (Cat: 21450, Thermo Scientific), according to the manufacturer’s protocol. Biotinylation of the antibody was measured using the Pierce Biotin Quantitation Kit (Cat: 28005, Thermo Scientific). Subsequently, 200 μl Exo-Flow streptavidin-coated magnetic beads that had been washed twice with Wash Buffer, were incubated with 10 μg of biotinylated BIN1 (Cat: sc-13575, Santa Cruz) or 10 μg biotinylated anti-Tau antibody HT7 (Cat:MN1000B, Thermo Fisher) for 2 h on ice. Subsequently, antibody-conjugated beads were washed 3 times with Wash Buffer. Concentrated CSF (Five milliliters of CSF that was concentrated to approximately 100 μl with Amicon 100KDa 15 ml centrifugal filters (Cat: UFC90102, Millipore)) was diluted 1:5 in Wash Buffer to a final volume of 500 μl, added to the antibody-conjugated beads, and incubated on a rotating rack at 4 °C overnight for capture. The next day, antibody-conjugated beads and captured EVs were washed twice with Wash Buffer and eluted from the beads with 20 μl of Elution buffer. Recovered EVs were lysated by adding 20 μl of Lysis Buffer (as described below), heated at 70 °C for 10 min, and loaded on gel for western blot.

Differential ultracentrifugation was performed as reported previously^40^. Briefly, conditioned media from 293T cell cultures were subjected to successive centrifugations at increasing speeds: 300 g for 10 min, 2,000 g for 10 min, and 10,000 g for 30 min to eliminate dead cells and large debris. After each centrifugation, the pellet was discarded and the supernatant was used in successive steps. The final supernatant was centrifuged at 100,000 g for 120 min, which yielded extracellular vesicles in the pellet. The pellet was washed with ice-cold PBS to eliminate free-floating non-EVs-associated proteins, and then centrifuged at 100,000 g for 120 min. The pellet containing EVs was resuspended in PBS or Lysis Buffer depending on the downstream analysis.

Izon Columns (qEV single column (iZON)) were used to purify EVs from conditioned media from primary microglia according to the manufacturer’s protocol. One hundred microliters of concentrated conditioned media had been loaded on the column and 1 ml of void volume was discarded followed by collection of two fractions of 500 μl each. These fractions were concentrated using an Amicon 10KDa filter (as described above), pooled and used for western blot analysis^72^.

### Nanoparticles tracking analysis

Nanosight LM 10 and NS300 devices (Malvern) were used according to the manufacturer’s instruction. The settings were determined before each session as average condition among the samples and fixed for all the measurements during the session. The LM10 was used with the following settings: laser Red, temperature 22 °C, camera level 16, camera shutter/ms 30, camera gain 680, while the NS300 was used with the following settings: laser Blue488, temperature 25 °C, camera level 13, shutter/ms 30, camera gain 250. For each sample, 3 to 5 videos of 30 seconds with at least 10 particles/frame were captured. Analysis was performed with NTA 3.2 software with the default settings. Results include all videos acquired for a given sample.

### Immuno electron-microscopy

Five microliters of PBS containing EVs in suspension was deposited on Formvar/carbon coated grids for 1 min. Then, the grid was moved to a drop of double distilled water and excess liquid was removed with filter paper. The sample was made hydrophilic by a 30 sec exposure to a glow discharge, and blocked in 1% BSA for 10 min. Subsequently, the sample was incubated for 30 min with 5 μl of Bin1 antibody (Cat: sc-13575, Santa Cruz), or IgG in 1%BSA. After washes in PBS, the sample was incubated for 30 min in Rabbit anti-mouse bridging antibody in 1%BSA (Cat: Ab6709, Abcam, 1:75 dilution) for 20 min. Following washes in PBS, the sample was incubated for 20 min in Protein A-gold (10 nm) in 1%BSA (University Medical Center Utrecht, the Netherlands, 1:50 dilution) and washed twice in PBS and 4 times washes in water. The sample was then stained by floating it on a small drop of 0.75% uranyl formate for 30 sec. After removing the excess uranyl formate with a filter paper, the grids were examined using a TecnaiG² Spirit BioTWIN, and images were recorded with an AMT 2k CCD camera at a primary magnification of 20,000–50,000×. Procedures were performed by Dr. Maria Ericsson at the Harvard Electron Microsopy Facility at Harvard Medical School, Cambridge, MA, USA. Materials purchased at EMS www.emsdiasum.com or Ted Pella www.tedpella.com. The number of anti-BIN1^+^ or anti-IgG^+^ EVs were manually scored from 5–15 images/condition, in 3 independent experiments, each of which was the product of pooling the CSF samples from 3–4 individuals.

### Plasmids

pLVX Ires-Neo lentiviral plasmid from Clontech was cut with EcoRI/MluI to remove the IRES-neo cassette. GFP-P2A linker expression cassette with a new multiple cloning site was synthesized as linear dsDNA (gBLOCK (IDT)) and cloned into the cut vector using NEB Builder DNA assembly master mix (New England Biolabs). All Bin1 isoforms were then synthesized as gBLOCKs (IDT) and cloned into the GFP-P2A lentiviral vector using NEBuilder DNA assembly master mix, according to the manufacturer’s protocol. pLVX Ef1a Ires puro vector from Clontech was cut with EcoRI/SpeI. Plasmids expressing human Tau 2N4R (Cat#: RC213312) and murine Bin1 transcript variant 2 (Cat#:MR207643) are commercially available (Origene). An insert encoding 2N4R human Tau with a P301L mutation and YFP at the C-terminus was synthesized as a linear DNA string, and cloned into pLVX Ires-Puro lentiviral plasmid from Clontech using NEB HIFI DNA assembly kit. 2N4R human Tau with a P301S mutation was PCR amplified and cloned into pSBI-CMV-SmBiT-Ires-Hygro, using NEB Builder HIFI DNA Assembly master mix.

### Recombinant tau expression and purification

His-hTau 2N4R or myc-hTau 2N4R P301S were overexpressed in BL21 Star (DE3) pLysS bacteria and purified as previously described^73^. Briefly, pelleted cells were resuspended on ice in high-salt RAB buffer pH 7 (0.1 M MES, 1 mM EGTA, 0.5 mM MgSO_4_, 0.75 M NaCl, 0.02 M NaF, 1 mM PMSF, 0.1% protease inhibitor cocktail (100 μg/ml each of pepstatin A, leupeptin, TPCK, TLCK, soybean trypsin inhibitor and 100 mM EGTA), homogenized, boiled at 100 °C for 10 min and then rapidly cooled on ice for 20 min. After centrifugation at 70,000 g for 30 min, the supernatant was dialyzed into FPLC Buffer (20 mM piperzine-N,N′-bis (2-ethanesulfonic acid), 10 mM NaCl, 1 mM EGTA, 1 mM MgSO_4_, 2 mM DTT, 0.1 mM PMSF pH 6.5), and applied to a 5 mL HiTrap Sepharose HP IEX cation-exchange (SP sepharose) column (GE Healthcare). Tau was eluted using a 0- to 400 mM NaCl gradient in FPLC Buffer. Peak fractions were pooled and dialyzed into 100 mM sodium acetate pH 7, and concentrated to approximately 5 mg/ml using an Amicon spin concentrator (Millipore). Final protein concentration was determined using bicinchoninic acid protein assay (BCA, Pierce).

### Preparation of Tau Fibrils

Tau protein was diluted to 40 µM in 100 mM sodium acetate pH 7 with 2 mM DTT and heated for 10 min at 55 °C. Low molecular weight heparin (Sigma) was added to 40 µM. The mixture was distributed to a 96-well plate and shaken in a tabletop Thermomixer (Eppendorf) at 37 °C and 1,000 rpm for 7 days. Insoluble Tau was pelleted by ultracentrifugation at 100,000 g for 30 min at 4 °C. The pellet was washed twice, with resuspension by vigorous pipetting in 100 mM sodium acetate pH 7. Aggregate formation was monitored by thioflavin assay and electron microscopy.

### Cell transfection

HEK293T were maintained in DMEM (Cat:11965-092, Life Technologies) supplemented with Exosome-depleted 10%FBS (Cat: A25904DG Life Technologies) and penicillin/streptomycin (Cat:15140-122, Life Technologies). Cells (2.5 * 10^6^/condition) were plated in 10 cm dishes and transfected with a total of 12 μg of vector expressing Tau 2N4R, Tau 2N4R P301L-YFP, Tau 2N4R P301S, human BIN1 isoform 1, murine Bin1 transcript variant 2, or human BIN1 isoform 9 using 36 μl Lipofectamine 2000 (Cat:5653, Life Technologies) in Optimem (Cat:31985-070, Life Technologies). After 48 hours, conditioned media as well as cells were harvested. Media was spun at 2,000 g for 10 min at 4 °C, and then at 10,000 g for 30 min at 4 °C. EVs were subsequently purified by Differential Ultracentifugation as described above. Protein lysate was prepared from 1 × 10^6^ 293T cells by adding 100 μl of Lysis Buffer (140 mM NaCl, 10 mM HEPES pH7.4, 1% NP-40) plus 1 mM PMSF, 1 mM DTT, Complete Protease Inhibitor Cocktail (Cat:11836170001, Roche). After 1 h incubation on ice, lysates were cleared by spinning at 13,000 rpm for 10 min at 4 °C. Protein concentrations were determined by BCA protein assay (Cat: 23228, Thermo Scientific).

### Primary microglia

Primary mixed glia cell culture was prepared from the cerebrum of P0 pups. Briefly, cortices from pups were removed from the skull and transferred to a 60 mm dish with HBSS and 0.37% Glucose and then minced with sterile blade. After adding Trypsin 10X, cortices and incubated at 37 °C in 5%CO_2_ for 20 min. Subsequently, complete DMEM (containing 10%FBS and Pen/Strep) was mixed with the cell suspension and transferred to a 15 ml tube and spun at 200 g for 5 min at 20 °C. After removing the supernatant, the cell pellet was resuspended in complete DMEM and plated in a T25 flask, where cells were maintained in the presence of murine GM-CSF (2 mg/ml) (Cat: 215-GM, R&D Systems). Media was replaced every 3 days. After 10 days, flasks were shaken at 50 rpm for 30 min at 37 °C and floating microglial cells were collected and pooled together by genotype. Cells were counted and plated at 1–2 × 10^6^ in 10 cm Primaria plates (Cat:353803, Corning). The following day, primary-cultured murine microglia were incubated with 10 μg/ml of recombinant human Tau for 3 h at 37 °C with 5%CO2. The cells were washed twice with DMEM and stimulated with LPS (from *E*. *coli* 0111: B4, 1 μg/ml, cat: L3024, Sigma-Aldrich) for 3 h. After the LPS stimulation, media was removed and the cells were treated with 5 mM ATP (Cat: A6559-25UMO, Sigma-Aldrich) for 15 min. The conditioned media was then collected, spun at 2,000 g for 10 min at 4 °C, and subsequently at 10,000 g for 30 min at 4 °C, and then resulting EVs were purified by commercial Sepharose Size-Exclusion Chromatography (IZON, MA) as described above. Microglia cells were washed in PBS and lysed in Lysis Buffer (as described above). After 1 h incubation on ice, lysates were cleared by spinning at 13,000 rpm for 10 min at 4 °C. Protein concentrations were determined by BCA protein assay.

### Organotypic brain slices culture

Brains were dissected from decapitated postnatal p2-p4 C57BL/6 J pups (Cat: 000664, The Jackson Laboratories) and glued (Glue Loctite) to the chuck of a water-cooled vibratome (Leica VT1000A) and trimmed with a commercial shave razor. Under aseptic conditions, 300 μm-thick coronal sections were sliced and collected in sterile medium. The organotypic slices were then placed in a 0.4-μm membrane insert (Millipore PICM03050) in a 6-well plate and cultured in presence of Basal Medium containing HBSS 1X, 15% Horse serum, GlutaMax, 45% Glucose, Pen-strep, N2 supplement, and fresh PDGF (10 ng/ml) and maintained at 35 °C and 5% CO_2_. Slices were infected with the respective AAV after 3 days of culturing. After AAV infection, slices were cultured for 9–12 days, during which time media was replenished with half media changes every three days. Slices were collected and lysed with Lysis Buffer as described previously.

### Western blots

All samples were lysed in Lysis Buffer, as described previously. Concentrated CSF Sepharose Size-Exclusion Chromatography purified EVs, shown in Fig. 1A,B, were mixed with Lameli Buffer (cat:161-0747, Biorad) + β-mercaptoethanol, boiled at 99 °C for 10 min and run in custom made Tris-Glycin SDS 7.5% gel (30% Acrylamide/Bis (Cat:1610156, Biorad), 1.5 M Tris HCL pH:8.8, Resolving gel buffer (Cat: 161-0798, Biorad), 0.5 M Tris HCL pH:6.8, Stacking gel buffer (Cat: 161-0799, Biorad), TEMED (Cat: 161-0800, Biorad), 10%APS (Cat: 161-0700, Biorad), 10% SDS (Cat: 71736-100, Sigma), dH_2_0) with Tris-Glycin SDS running buffer (Cat:161-0732, Biorad). BIN1 and Tau IPs, shown in Fig. 2A,B, were mixed with LDS buffer (Cat: B0007, Life Technologies), heated at 70 °C for 10 min and run on NuPage Bis-Tris 4–12% gel in MOPS Running Buffer (Cat:NP0001, Life Technologies). SEC purified EVs (Supplementary Figs 1 and 2), 293T cell lysates and CM EVs (Fig. 3A,B and Supplementary Fig. 8), primary microglia cell lysates and CM EVs (Fig. 5B) and Organotypic Brain Slices (Supplementary Figs 4 and 6) were mixed with LDS buffer, heated at 70 °C for 10 min and run on NuPage Tris-Acetate 7% gel using Tris-Acetate SDS Running Buffer (Cat log: LA0041, Life Technologies). As control, EV purified from plasma of healthy donors was used (Cat: HBM-PEP-30, Hansa BioMed).

All the gels were run at 120 V for 2 h and then transferred to a Nitrocellulose membrane (iblot 1B23001, Life Technologies) with iblot-2 (Thermo Scientific). After 1 h blocking in 5% (w/v) non-fat milk in TBS with 0.1% Tween 20, the membranes were then incubated overnight at 4 °C with the following antibodies diluted 1:500 in TBS with 0.1% Tween 20: Bin1 99D (sc-13575, Santa Cruz), Bin1 (Ab153912, Abcam), Tau13 (835201, Biolegend), Tau22 (ABN454, Millipore) previously validated^42^, AT270 (MN1050, Thermo Scientific), Tsg101 (Ab30871, Abcam), Cd9 (Ab92726, Abcam), GAPDH 1:2000 (Ab189095, Abcam), Clathrin (P1663, Cell Signaling), Alix (ABC40, Millipore), β-actin (Ab8229, Abcam). The membranes were washed in TBST (4 × 10 min) before 1 h incubation with True-Blot anti-rabbit (Cat: 18-8816-33, Rockland) or True-Blot mouse (Cat:18-8817-33, Rockland) conjugated HRP at 1:1000 dilution to avoid detection of non-specific bands. The immunoblots were then visualized using ECL (Cat: 32209, Thermo Scientific). Images were acquired with Amersham Imager 600 (GE Healthcare Bio-Sciences, PA).

### Generation of FRET assay cell line

Tau RD domains (residues 243-375) carrying a P301S mutation with C-terminal CFP and YFP fusions were cloned with a P2A linker into pLVX Ef1a Ires Puro lentiviral vector (Cat: 631987, Clontech). Lentiviral particles were generated using Virapower lentiviral packaging mix (Cat: K497500, Life Technologies) following the manufacturer’s protocol. In short, HEK 293 T cells were transfected with Tau expressing plasmid and Virapower packaging mix. Forty-eight hours later viral particles in the media were harvested, passed through a 0.45 μm filter, and then added to newly plated HEK293 cells in the presence of 5 μg/ml polybrene. Cells were centrifuged at room temperature 700 g for 2 hours. The next day transduced cells were selected with 2 μg/ml puromycin. The pool of stable cells generated were single-cell cloned by limiting dilution and the most responsive clones to presence of Tau fibrils were isolated for further experiments.

### FRET assay

HEK-TauRD P301S FRET cells were plated at 25,000 cells/well in 96-well PDL coated plate (Cat: 354461, Corning) in growth media (MEM, 10%FBS, Pen/Strep, Puromycin 2 μg/ml). The day after, BIN1 affinity-purified EVs in 100 μl of Elution Buffer or 100 μl of BIN1-immunodepleted CSF diluted in Wash Buffer was mixed with 80 μl of Optimem with 20 μl of Lipofectamine 2000, and incubated at room temperature for 10 min. Subsequently, growth media was removed from the cells, replaced with samples containing Lipofectamine, and incubated at 37 °C, 5%CO2. After 1 h, Lipofectamine-containing media was removed from the cells and replaced with growth media. Cells were maintained in culture at 37 °C, 5%CO2 for 72 h afterward. The day of the analysis, cells were washed in PBS, detached with Trypsin 0.25%, and washed with FACS buffer (PBS + 2% FBS). Subsequently, cells were fixed in 2%PFA, 2% Sucrose for 15 min at 4 °C, spun at 12,000 rpm for 15 min at 4 °C, resuspended in FACS buffer and acquired with a 5 lasers system LSRII (Becton Dickinson), using pacific-orange and pacific-blue dyes for YFP and CFP, respectively. Data was analyzed with FlowJo and expressed as Integrated FRET Density.

### Mouse lines

Mouse strains B6.129S6-Bin1tm2Gcp/J (Bin1flox/flox), B6J.B6N(Cg)-Cx3cr1tm1.1(cre)Jung/J (Cx3Cr1-Cre) and B6; C3-Tg (Prnp-MAPT*P301S)PS19Vle/J (PS19) were purchased from Jackson Laboratories (cat. No. 021145, 025524 and 008169, respectively). Mice were housed with a 12 h light/dark cycle, and food and water were provided *ad libitum*.

### Stereotaxic injection

Mice were given Buprenorphine Animalgesics (sustained release, 3.25 mg/kg s.c.) and anesthetitization with Isoflurane (first in a chamber (3% mixed with oxygen) and then maintained with a nose cone (1.25% maintenance). After mice were immobilized in a stereotaxic apparatus, an incision was made over bregma, and a burr hole was drilled through the skull. The study reagent was injected using a motorized microinjector and a 10 μl gas-tight Hamilton microsyringe at rate of 0.5 μl/min. The needle was allowed to remain for 2–4 minutes following injection to prevent reflux of fluid and then slowly removed over 2 minutes. After needle removal, the skin incision was closed with wound glue. Mice were monitored daily for a minimum of 7 days to ensure proper recovery and absence of infection. Starting at 6 months of age, mice were weighed and monitored weekly, following guidelines reported below.

### AAV injection in PS19 mice

Recombinant AAV serotypes 1 and 8 (1:1) expressing human BIN1 isoform 9 were generated by cloning human BIN1 isoform 9 from pLVX-GFP-P2A-hBIN1isoform9 (described above) under the control of CAG promoter. Recombinant AAV serotypes 1 and 8 (1:1) expressing human BIN1 isoform 1 were generated by cloning human BIN1 isoform 1 from pLVX-GFP-P2A-hBIN1isoform1 (described above) under the control of hSYN promoter. AAV(1N8)-CAG-GFP-P2A-hBIN1isoform9, AAV(1N8)-CAG-GFP-U6-scrmb-shRNA, AAV(1N8)-hSYN-eGFP-P2A-hBIN1isoform1 and AAV(1N8)-hSYN-eGFP-WPRE were prepared and CsCl-purified by Vector Biolabs (https://www.vectorbiolabs.com). Male PS19 mice at 3 months of age received a single stereotaxic injection of 1.5 μl of AAV(1N8)-CAG-GFP-P2A- hBIN1isoform9 (1.9 × 10^13^ GC/ml) or AAV(1N8)-CAG-GFP-U6-scrmb-shRNA (5.3 × 10^13^ GC/ml) at hippocampal coordinates: bregma antero-posterior 2.5 mm, medial-lateral −1.7 mm, and dorso-ventral 1.6 mm, following the procedure described above. Starting at 6 months of age, mice were weighed and started to be monitored weekly, following guidelines reported below. When reaching Clinical score 2, the mouse was euthanized, and intracardially perfused with saline solution. The brain was collected and fixed in Neutral Formaline Buffer (Cat: 5701, Thermo Fisher). Alternatively, male PS19 mice at 3 months of age received a single stereotaxic injection of 1.5 μl of AAV(1N8)-hSYN-eGFP-P2A- hBIN1isoform1 (3.5 × 10^12^ GC/ml) or AAV(1N8)-hSYN-eGFP-WPRE (3.8 × 10^12^ GC/ml) at hippocampal coordinates: bregma antero-posterior 2.5 mm, medial-lateral −1.7 mm, and dorso-ventral 1.6 mm, following the procedure described above. Between 250 and 300 days of age, mice were euthanized, intracardially perfused with saline solution, brains collected and fixed in Neutral Formaline Buffer (Cat: 5701, Thermo Fisher).

### Tau Fibrils injection in Bin1^flox/flox^:: Cx3Cr1-Cre;PS19

Human Tau containing 2N4R, the P301S mutation and myc-tagged Tau was produced, purified and stored at −80 °C (described above). Fibrils were generated by incubating monomers with heparin 40 µM in 100 mM sodium acetate pH 7 at 37 °C for 5 days. The fibril suspension was centrifuged, and the pellet was resuspended in PBS to a final fibril concentration of 2 mg/mL (based on monomer equivalents). The Tau Fibrils were sonicated (Elmasonic S 40/(H), ultrasonic frequency: 37 kHz) at RT, before stereotaxic injection at hippocampal coordinates: bregma antero-posterior −2.0 mm, medial-lateral 1.6 mm, and dorso-ventral −1.7 mm, following the procedure described above. Bin1^flox/flox^:: Cx3Cr1-Cre^**+**^:: PS19^**+**^ and control littermates Bin1^wt^:: PS19^**+**^ (a mix of Bin1^flox/wt^:: Cx3Cr1-Cre^**−**^:: PS19^+^ and Bin1^flox/flox^:: Cx3Cr1-Cre^**-**^:: PS19^+^) mice received a single stereotaxic injection of 2 μl (2 μg) at 2–3 months of age. 90 days post-surgery, mice were euthanized, perfused, and brains fixed as above.

### PS19 monitoring

Mice expressing Tau P301S (PS19) show onset of symptoms around 6 months of age^44^. Thus, we implemented a system of monitoring based on the following Clinical scoring (CS): CS 0 Normal; CS 1 Abnormal gait (low walk with good mobility in cage), able access food and water and/or body weight loss (BWL) ≈10%; CS 2 Mild hind limb weakness/paresis effecting both or one leg, hind limb muscle wastage noted. Good mobility in cage and/or 10% < BWL ≤ 20%; CS 3 Moderate hind limb paresis with or without front limb weakness/paresis, able to right on both left and right side within 15 seconds when placed lateral recumbent and/or 20% < BWL < 32%. Fair mobility in cage; CS4 Severe hind limb paresis with or without front limb weakness/paresis, not able to right when placed lateral recumbent on either the right or left side, and/or paralysis in any limbs and/or body weight loss ≥32%. Poor mobility in cage.

### Immunohistochemistry

Tissue preparation: mice were cardiovascularly perfused with PBS and brains were fixed in 10% neutral-buffered formalin for 72–96 hours (Cat: 032–059, Fisher Scientific). The right side of each brain was marked by ink. Brains were dissected coronally into 3 or 6 slabs, processed and blocked on a Tissue Tek VIP tissue processor (Sakura Finetek) and embedded into paraffin in an anterior face down orientation. Five-micron thick paraffin sections were generated either manually (using a Leica rotary microtome RM2255) or on an automated slide preparation instrument AS-400 (Dainippon Seiki, Japan). Serial 5-micron sections were collected from each sample and placed on charged slides. For each sample, 2 or 3 sections, separated by approximately 200–250 micron, were subjected to immunohistochemistry analysis. The following antibodies were used: chicken polyclonal anti-GFP (Cat: ab13970, Abcam, final concentration 2 μg/ml), rabbit polyclonal anti-chicken (Cat: 303-005-003, Jackson Labs), human monoclonal anti-phospho-Tau (phospho-Ser 202/phospho-Thr 205, generated internally at Biogen, Clone 40E8, final concentration 0.07 μg/ml, previously validated^47^), biotinylated anti-Human IgG (Cat: BA-3000, Vector Labs), mouse monoclonal anti-amphiphysin II (BIN1) antibody conjugated to FITC (Cat: sc-23918FITC, Clone 2F11, Santa Cruz, final concentration 0.028 μg/ml), rabbit polyclonal anti-FITC (Cat: A11090, Life Techologies). Immunohistochemistry staining was performed on an automated Ventana Discovery Ultra platform. Briefly, slides were baked at 60 °C and deparaffinized. Heat-mediated epitope retrieval was performed in the Ventana CC1 retrieval solution (pH 8.0) and then slides were incubated with 3%H_2_O_2_ to quench the endogenous peroxidase activity. Slides were incubated with respective primary antibody solutions for 60 min, followed by and secondary antibodies if applicable. Thereafter, slides were incubated with polymer horseradish peroxidase-conjugated antibodies (Ventana), followed by addition of DAB. Slides were counterstained with hematoxylin, dehydrated and mounted.

For immunofluorescence labeling of AAV infected cell types, deparaffinized hippocampal sections prepared as above were incubated with primary antibodies overnight at 4 °C in PBS with 0.3% Triton-X and 10% Normal Donkey Serum (Jackson Immunoresearch). After three PBS washes, sections were incubated for 90 minutes with species-specific cross-absorbed secondary antibodies (Jackson Immunoresearch), washed in PBS, and coverslipped with Fluoromount containing DAPI (Southern Biotech). Antibodies included: chicken polyclonal anti-GFP (Cat: ab13970, Abcam, final concentration 10 μg/ml), rabbit polyclonal anti-Iba1 (Cat: 019-19741,Wako, final dilution 1:1000), rabbit polyclonal anti-GFAP (cat:NB300-141, Novus, final dilution 1:1000), rabbit polyclonal anti-NeuN (cat: ab104225, Abcam, final concentration 1 μg/ml), guinea pig polyclonal anti-NeuN (cat: ABN90, Millipore, final dilution 1:1000). Immunofluorescent labeling of BIN1 to validate a microglial selective deletion of BIN1 in the cKO mice had been attempted by testing several commercial anti-Bin1 antibodies. Unfortunately, none of the tested Abs demonstrated a specific signal in mouse brain.

### Imaging and analysis

Stained slides were scanned at 20x magnification on a 3DHistech Panoramic P250 Slide scanner. Embedding pathology marker dye was used to confirm correct left/right orientation on digital images. Images were analyzed with custom image analysis algorithms using Visiopharm image analysis software. Images were manually annotated by blinded analyst bilaterally on all slides. Annotated regions (hippocampus and entorhinal Cortex) were analyzed based on Visiopharm’s HDAB-DAB filter to detect pTau, BIN1 or GFP staining. Relative area of pTau, BIN1 or GFP was calculated as a percent of total tissue area in annotated regions. Relative percentage of phospho-Tau IHC staining area was calculated by dividing the area of positively stained area by the total annotated region area.

For immunofluorescence analysis of AAV infected cell types, tiled full thickness 20x images of the entire hippocampus were obtained on a Zeiss Axio Observer using ZEN software. Overlap between GFP-expressing cells and GFAP, NeuN, or Iba1 cell-type markers was manually scored by a blinded observer. Densitometric analysis of WB bands was performed with ImageJ, NIH.

### Microglia sorting

Briefly, animals were anesthetized with Ketamine/Xylazine i.p. injection (1:1, concentration) and ice-cold PBS was used for perfusion and brains collection. After removing Cerebellum and Olfactory bulbs, remaining brain tissue was minced in Petri dish and transferred to 15 ml tube with 3 ml of Accutase (Cat: SCR005, Millipore); and incubated at 4 °C for 30 min in a rotary shaker. Then, large tissue chunks were allowed to settle at the bottom by gravity, and the cell suspension from the top of the tube were passed through pierce tissue strainer (Cat:87791, Thermo Fisher) and collected in fresh tubes. Two ml of HBSS (Cat:14185-052, Life Technologies)/HEPES (Cat:15630-080, Life Technologies) buffer was added to the chunk tissues and triturated with 5 ml pipette until almost homogenous. Following gravity separation, supernatants were passed through a pierce tissue strainer and pooled to the same tube containing cell suspension from previous step. Trituration was repeated with the remaining tissue chunks with a 1 ml pipette until completely homogenous and passed through a pierce strainer and pooled to the rest of the cell suspension. Then, the strainer was rinsed with HBSS/HEPES buffer to bring the cell suspension to 15 ml, prior to a spin at 600 × g for 5 min at 4 °C (brake 5). The supernatant was aspirated, and the pellet was resuspended with 1 ml of 100% FBS. Nine milliliters of 33% Percoll (Cat:17089102, GE Life Science) was added to the cell suspension and mixed well. One milliliter of 10%FBS was overlayed on the Percoll/cell suspension and spun at 800 g at 4 °C for 15 min (break 1 or lowest possible). The supernatant was aspirated without disturbing the pellet, and the pellet was resuspended with 1 ml of FACS buffer (HBSS, 1%BSA, 2 mM EDTA, 25 mM HEPES, 0.09% NaN_3_). Additional FACS buffer was added to bring the cell suspension to 15 mL before spinning at 600 g for 10 min at 4 °C. Following centrifugation, the pellet was resuspended with 300 μl of FACS buffer and filtered through 30 μm filter (Partec Celltrics, 04-004-2326) for further analysis.

### Cell staining for sorting

Filtered cells were pelleted down with gentle centrifugation and incubated with 50 μl of Fc blocker anti-mouse CD16/32 (Cat:101321, Biolegend) on ice for 10 min. Then 50 μl of CD11b-PE Rat anti-mouse antibody (Cat: 553311, BD bioscience) and CD45 BV421 anti-mouse antibody (Cat: 563890, BD bioscience) were mixed with the cells and incubated for 30 min on ice. Following incubation, cells were washed and resuspended in 200 μl of FACS buffer. Cells were filtered through a 30 μm filter before FACS sorting (BD FACS Aria Fusion) and collecting approximately 20,000-100,000 CD11b^+^; CD45^med^ cells in FACS buffer. Unstained and single-stained cells were used as controls for gating the cells in FACS. Sorted cells were centrifuged at 6800 g for 15 min and the cell pellets were snap frozen and stored at −80 °C until RNA extraction.

### RNA extraction

Qiagen All Prep kit (Cat: 80224, Qiagen) was used for RNA extraction from previously frozen microglia cells. Briefly, cells were lysed with RLT Buffer (with β-mercaptoethanol added), and passed through QIA shredder (Cat: 79654, Qiagen). Flow-through from the QIA shredder was then passed through a DNA Mini column to remove the DNA from the lysate. DNA mini columns were stored at 4 °C for DNA purification later; and the passed-through lysates were treated with proteinase K. Following treatment, 100% ethanol was added to the lysates and passed through a RNeasy Mini spin column. In-column DNase treatment was also performed. Subsequently, RNA was eluted with RNase-free water, quantified in Nano-drop and stored at −70 °C until samples were submitted for sequencing.

### RNA sequencing

cDNA from each sample was generated from 5 ng of total RNA using SMARTer Ultra Low Input RNA kit v4 (Clontech). The cDNA was amplified by 9 PCR cycles, followed by QC analysis on BioAnalyzer 2100 (Agilent). Sequence libraries were produced from 150 pg of cDNA using Nextera XT DNA library kit (Illumina), cleaned up with AMPure XP beads, and QC checked with Caliper LabChip GX. Paired-end sequencing data were generated on an Illumina HiSeq 2500, at a depth of 20 million reads per sample, with read length 50b × 2.

### RNASeq analysis

The reads were aligned with the OmicSoft OSA4 to the mouse genome (mm10) and the Ensembl.R84 gene model. Gene counts were estimated using RSEM. Six samples were removed (one from each cohort, except two samples from WT_Female cohort and zero samples from Heterozygote_Male cohort) due to low fraction of uniquely mapped paired reads and higher than expected duplication. Of note, these samples with poor technical QC metrics were also clear outliers in PCA. Normalization and differential expression analysis were carried out with the Bioconductor package DESeq 2. Differentially expressed genes (DEGs) were defined as having an average normalized alignment counts >4, adjpval < 0.05, and |FC| > 1.5. The lists of DEGs were analyzed for pathway and ontology enrichment using the Ingenuity IPA software.

### Geo Dataset analysis

Fastq files for GEO data set GSE89960 had been downloaded and analyzed in the same way as our internally generated RNASeq data, as outlined above. Alignment has been performed with STAR. Transcripts count were estimated using RSEM. TPM values were extracted for BIN1 gene as well as all reported transcripts. Fastq files for GEO data sets GSE52564 and GSE73721 were downloaded and analyzed in the same way as our internally generated RNASeq data, as outlined above. FPKM values were extracted for BIN1 gene ENSMUSG00000024381 as well as two of its transcripts, ENSMUST00000025239 and ENSMUST00000091967.

### Accession code

GSE132877.

### Statistical methods

Statistical analyses were performed using GraphPad Prism unpaired or paired parametric two-tailed Student’s t-test (95% confidence). D’Agostino & Pearson’s omnibus normality tests had shown that the distribution of the value in each experiment is normal. Tau spreading was assessed in the Contralateral Hippocampus by use of a 2-way ANOVA model with interaction for the factors of genotype and gender. The equal variance assumption of ANOVA was validated by conducting a Levene’s Test (p > 0.05) and normality was assessed with residual diagnostic plots. Contrasts of Heterozygous versus WT and Homozygous versus WT was assessed within each gender with the family-wise error rate controlled at 5% within the Contralateral Hippocampus via Holm’s p-value adjustment (two-sided). No outliers were identified or excluded. All experiments were performed as a minimum of 3 independent replicates. p-values < 0.05 were considered significant. No sample-size calculation had been performed. Sample-size used are similar to those generally employed in the field.

## Supplementary information

Supplementary Information

Supplementary Dataset 1

Supplementary Dataset 2
