# Supplementary material for: BIN1 favors the spreading of Tau via extracellular vesicles
Source: Sci Rep. 2019 Jul 1;9:9477. doi: 10.1038/s41598-019-45676-0 (PMC6603165; doi:10.1038/s41598-019-45676-0)
Supplement: Supplementary file 1 — Supplementary Information [file 41598_2019_45676_MOESM1_ESM.pdf]

BIN1 favors the spreading of Tau via extracellular vesicles

Andrea Crotti[1,*,#], Hameetha Rajamohamend Sait[1], Kathleen M. McAvoy[1], Karol Estrada[1], Ayla Ergun[2], Suzanne Szak[1], Galina Marsh[1], Luke Jandreski[1], Michael Peterson[1], Taylor L. Reynolds[1], Isin Dalkilic-Liddle[1], Andrew Cameron[1], Ellen Cahir-McFarland[1], Richard M. Ransohoff[3].

[1]Biogen, 225 Binney St., Cambridge, MA, 02142, USA

[2]Fulcrum Therapeutics, 26 Landsdowne St, Cambridge, MA, 02139, USA

[3]Third Rock Ventures, 29 Newbury Street, Suite 30, Boston, MA, 02116, USA

[*]andrea.crotti@astellas.com

[#] current address: Astellas, 1030 Massachusetts Avenue, Cambridge, MA, 02138, USA

Supplementary Figure 1

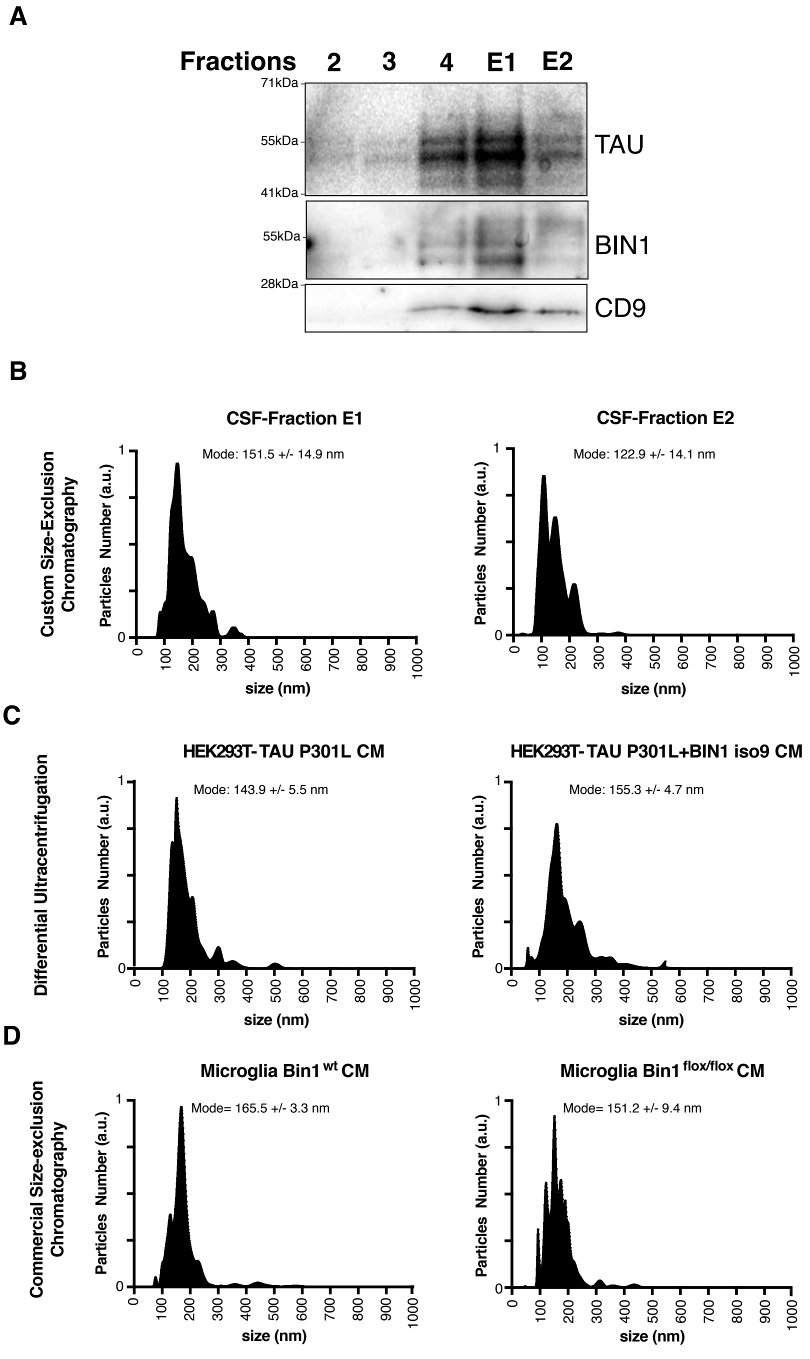

Supplementary Figure 1: **Analysis of EVs purified from human CSF and Cell Culture Conditioned Media with different methods.** (**A**) Five milliliters of pooled human CSF from individuals characterized as Braak's Stages III and IV were processed by Custom SEC as reported in the Material and Methods section. Each fraction containing extracellular vesicles was collected, concentrated, loaded on 7% Tris Acetate gel and analyzed for the presence of Tau, BIN1, and the exosome marker CD9 by WB. True-Blot secondary antibodies (Rockland) had been used to avoid detection of non-specific bands. WB images shown represent cropped sections of full blots shown in Supplementary Figure 14. One representative experiment shown out of 3 independent replicates. Fraction 1 is not shown because it represents void volume. (**B**) Nanoparticle tracking analysis of extracellular vesicles contained in fraction E1 and E2, obtained by Custom SEC fractionation of human CSF. (**C**) Nanoparticle tracking analysis of extracellular vesicles purified by Differential Ultracentrifugation from HEK293T Conditioned Media, after transfection with plasmids expressing Tau 2N4R P301L or Tau 2N4R P301L + BIN1 isoform 9, respectively. (**D**) Nanoparticle tracking analysis of extracellular vesicles contained in fraction 11-15 (pooled), obtained by Commercial SEC fractionation of primary microglia Conditioned Media. One representative experiment out of 3 independent replicates.

Supplementary Figure 2

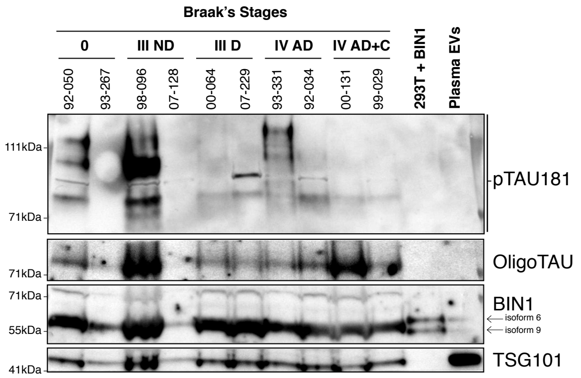

Supplementary Figure 2: **BIN1 and Tau are present in the EVs purified from CSF**. Western blot of EVs purified by SEC from CSF samples (reference codes reported) show the presence of BIN1, pTau181, oligomeric Tau and TSG101. As controls, EVs purified from Plasma of Healthy Donors and protein lysate from 293T cells co-transfected with plasmids expressing BIN1 isoform 6 and 9 were run as well (indicated by arrows). Plasma EVs show the presence of BIN1, as previously reported[34], as well as TSG101. True-Blot secondary antibodies (Rockland) had been used to avoid detection of non-specific bands. WB images shown represent cropped sections of full blots shown in Supplementary Figure 15.

Supplementary Figure 3

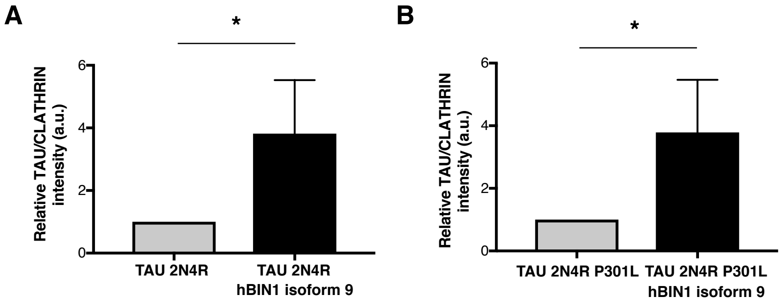

Supplementary Figure 3: **Densitometric analysis of Tau content in EVs purified from 293T cells.** Plots representing densitometric analysis of WB from EVs purified from CM of 293T cells transfected with Tau 2N4R alone or in presence of BIN1 isoform 9 (**A**) or 293T cells transfected with Tau 2N4R P301L-YFP alone or in presence of BIN1 isoform 9 (**B**). Tau band intensity is normalized to CLATHRIN band intensity. Plot represents mean±SD of 4 independent replicates. Paired parametric Student's t-test two tailed, 95% confidence: p-value: * p< 0.05; ** <0.01; *** p<0.001.

Supplementary Figure 4

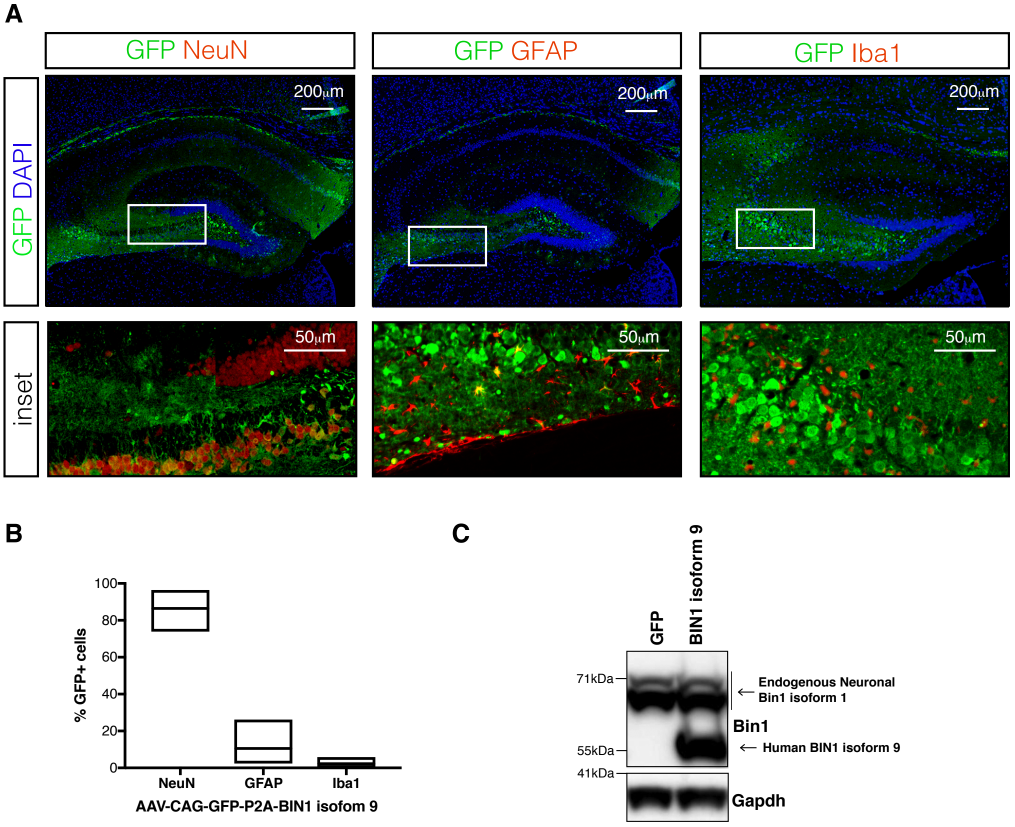

Supplementary Figure 4: **Analysis of AAV-CAG-GFP-P2A-hBIN1 isoform 9 infectivity. (A)** Immunofluorecence co-staining of GFP and cell type markers: NeuN (neurons), GFAP (astrocytes) and Iba1 (microglia) in hippocampi of PS19 mice injected with AAV-GFP-P2A-hBIN1 isoform 9. One representative image per group, scale: 200µm. Inset scale 50µm. (**B**) Plot representing the mean±SD percentage of GFP^{+} cells co-stained with NeuN, GFAP and Iba1, respectively, quantified on 8 animals. (**C**) Western blotting of organotypic brain slices infected with AAV expressing GFP or GFP-P2A-hBIN1 isoform 9. Arrows indicate endogenous neuronal Bin1 isoform 1 and human BIN1 isoform 9, respectively. One representative experiment of 3 independent replicates. WB images shown represent cropped sections of full blots shown in Supplementary Figure 16.

Supplementary Figure 5

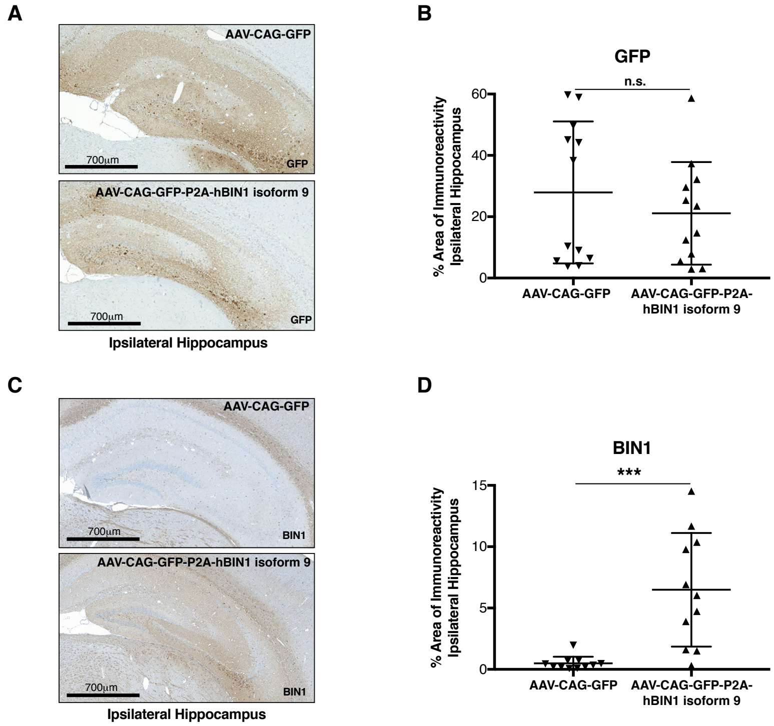

Supplementary Figure 5: **Immunohistochemical analysis of GFP and BIN1 staining in PS19 mice injected with AAV expressing GFP or GFP-P2A-hBIN1 isoform 9.** (A) Immunohistochemical staining of GFP in hippocampi of PS19 mice injected with AAV expressing GFP or GFP-P2A-hBIN1 isoform 9, respectively. One representative image per group. Scale: 700μm. (**B**) Plot representing the percentage of GFP staining (GFP area over total area of the hippocampus) for each animal. Each triangle represents one single animal. (**C**) Immunohistochemical staining of BIN1 in hippocampi of PS19 mice injected with AAV expressing GFP or GFP-P2A-hBIN1 isoform 9, respectively. One representative image per group. Scale: 700μm. (**D**) Plot representing the percentage of BIN1 staining (BIN1 area over total area of the hippocampus) for each animal. Each triangle represents one single animal. Unpaired parametric Student's t-test two tailed, 95% confidence: p-value: * $p< 0.05$; ** <0.01; *** $p<0.001$.

Supplementary Figure 6

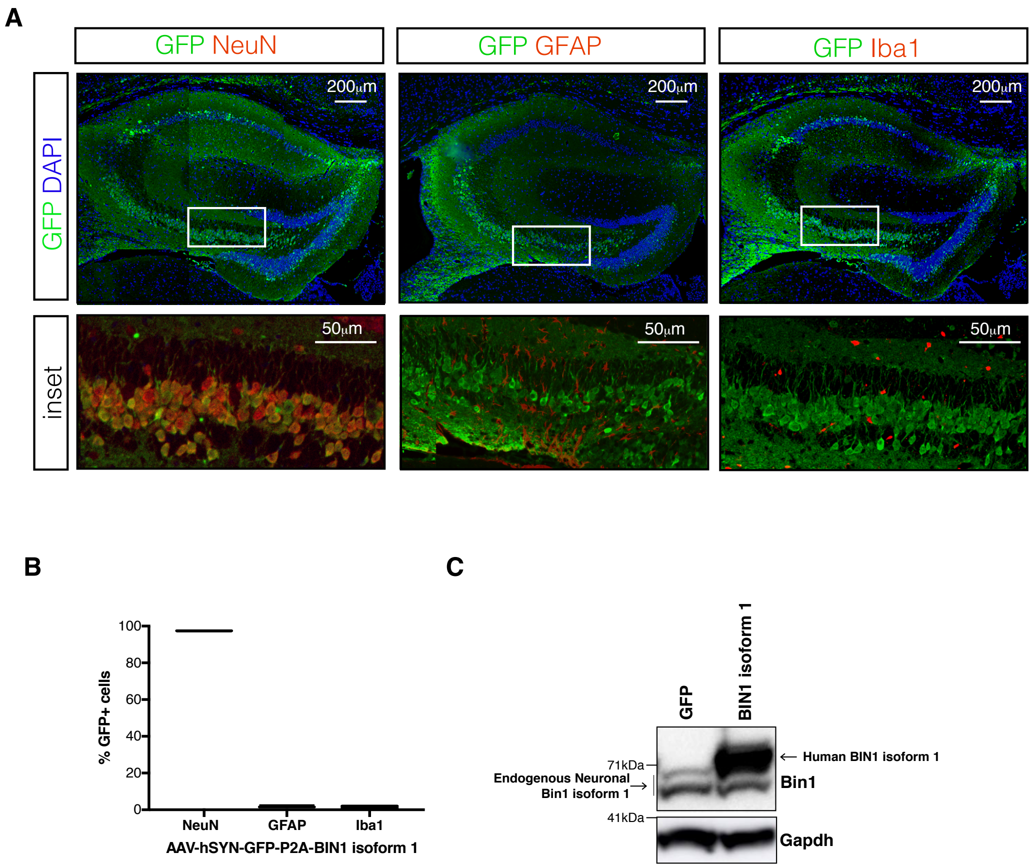

Supplementary Figure 6: **Analysis of AAV-hSYN1-GFP-P2A-hBIN1 isoform 1 infectivity.** (**A**) Immunofluorecence co-staining of GFP and cell type markers: NeuN (neurons), GFAP (astrocytes) and Iba1 (microglia) in hippocampi of PS19 mice injected with AAV-hSYN1-GFP-P2A-hBIN1 isoform 1. One representative image per group, scale: 200μm. Inset scale 50μm. (**B**) Plot representing the mean±SD percentage of GFP^{+} cells co-staining with NeuN, GFAP and Iba1, respectively, quantified on 4 animals. (**C**) Western blotting of organotypic brain slices infected with AAV expressing GFP or hSYN1-GFP-P2A-hBIN1 isoform 1. Arrows indicate endogenous murine Bin1 isoform 1 and human BIN1 isoform 1, respectively. One representative experiment of 3 independent replicates shown. WB images shown represent cropped sections of full blots shown in Supplementary Figure 17.

Supplementary Figure 7

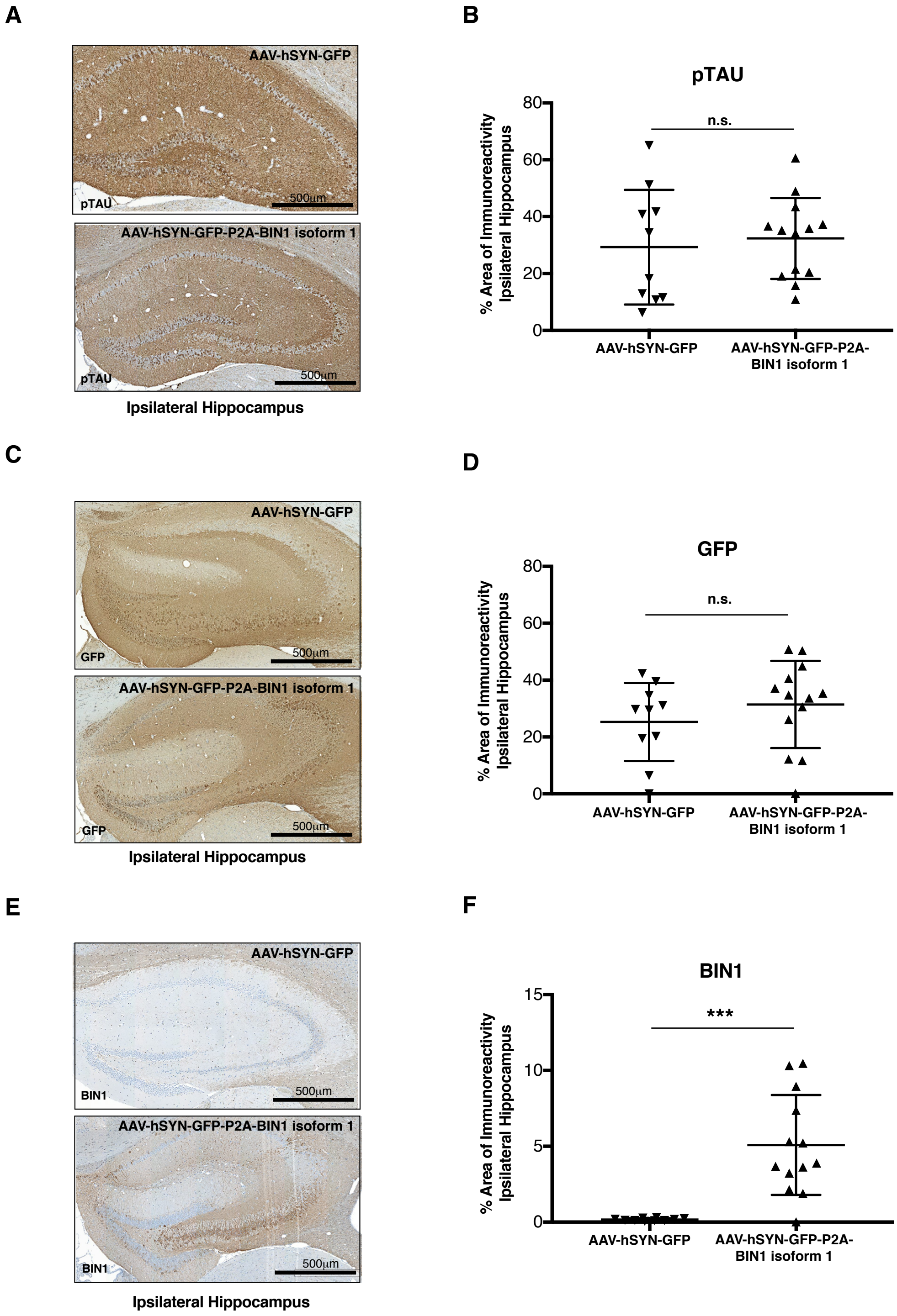

Supplementary Figure 7: **Immunohistochemical analysis of pTau, GFP and BIN1 staining in PS19 mice injected with AAV expressing GFP or hSYN1-GFP-P2A-hBIN1 isoform 1.** (**A**) Immunohistochemical staining of pTau in hippocampi of PS19 mice injected with AAV expressing GFP or hSYN1-GFP-P2A-hBIN1 isoform 1, respectively. One representative image per group. Scale: 500μm. (**B**) Plot representing the percentage of pTau Area of staining (pTau area normalized to total area of the hippocampus for each animal). Each triangle represents one single animal. (**C**) Immunohistochemical staining of GFP in hippocampi of PS19 mice injected with AAV expressing GFP or hSYN1-GFP-P2A-hBIN1 isoform 1, respectively. One representative image per group. Scale: 500μm. (**D**) Plot representing the percentage of GFP staining (GFP area normalized to the total area of the hippocampus for each animal). Each triangle represents one single animal. (**E**) Immunohistochemical staining of BIN1 in hippocampi of PS19 mice injected with AAV expressing GFP or hSYN1-GFP-P2A-hBIN1 isoform 1, respectively. One representative image per group. Scale: 500μm. (**F**) Plot representing the percentage of BIN1 staining (BIN1 area normalized to the total area of the hippocampus for each animal). Each triangle represents one single animal. Unpaired parametric Student's t-test two tailed, 95% confidence: p-value: * $p< 0.05$; ** <0.01; *** $p<0.001$.

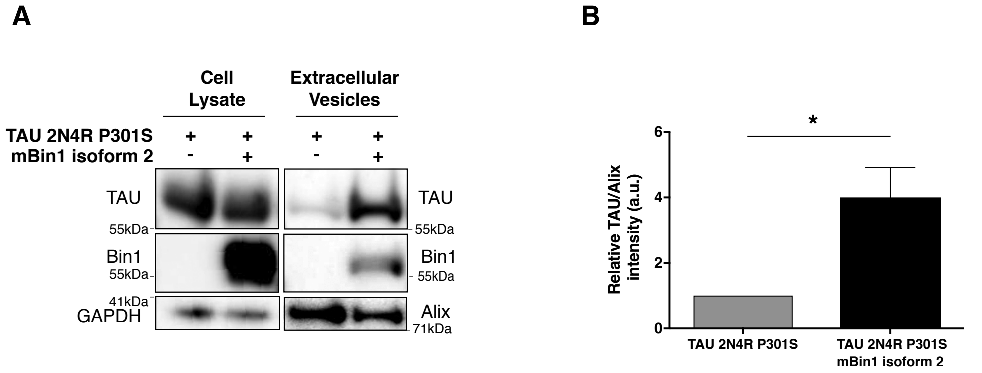

Supplementary Figure 8: **Murine Bin1 isoform 2 favors the release of Tau via EVs *in vitro*. (A)** Western blot of Tau and BIN1 in cell lysates (left panel) and EVs (right panel) from HEK293T cells transfected with Tau 2N4R P301S alone or in presence of murine Bin1 isoform 2. GAPDH (left panel) represents a cell lysate loading control. Alix (right panel) serves as an extracellular vesicle loading control. One representative experiment shown out of 3 independent replicates. WB images shown represent cropped sections of full blots shown in Supplementary Figure 18. **(B)** Plot representing densitometric analysis of WB from EVs purified from CM of 293T cells transfected with Tau 2N4R P301S alone or in presence of murine Bin1 isoform 2. Tau band intensity is normalized to Alix band intensity. Plot represents mean±SD of 4 independent replicates. Paired parametric Student's t-test two tailed, 95% confidence: p-value: * $p< 0.05$; ** <0.01; *** $p<0.001$.

Supplementary Figure 9

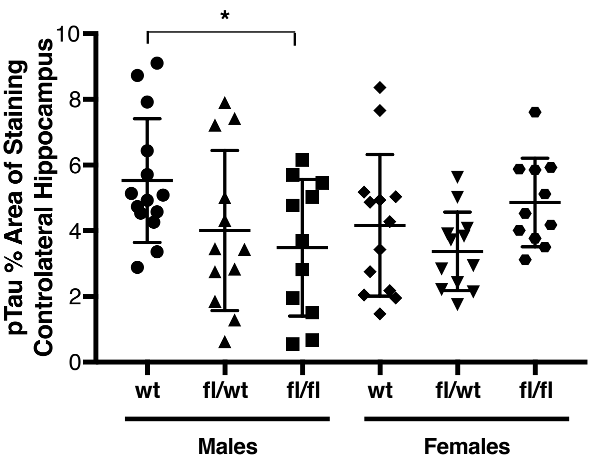

Supplementary Figure 9: **Deletion of Bin1 in microglia decreases pTau spreading *in vivo* in male but not female mice.** (**A**) Plot representing the percentage of pTau staining (area pTau reactivity of contralateral (non-injected) hippocampus normalized to the area of the contralateral hippocampus for each animal). Each symbol represents one single animal. (**B**) Plot representing the percentage of pTau Area of staining (Area pTau reactivity of ipsilateral injected hippocampus normalized to the area of the ipsilateral hippocampus for each animal). Each symbol represents one single animal. Two-way ANOVA with Holm's p-value adjustment (two-sided). p-value: * p< 0.05; ** <0.01; *** p<0.001.

Supplementary Figure 10

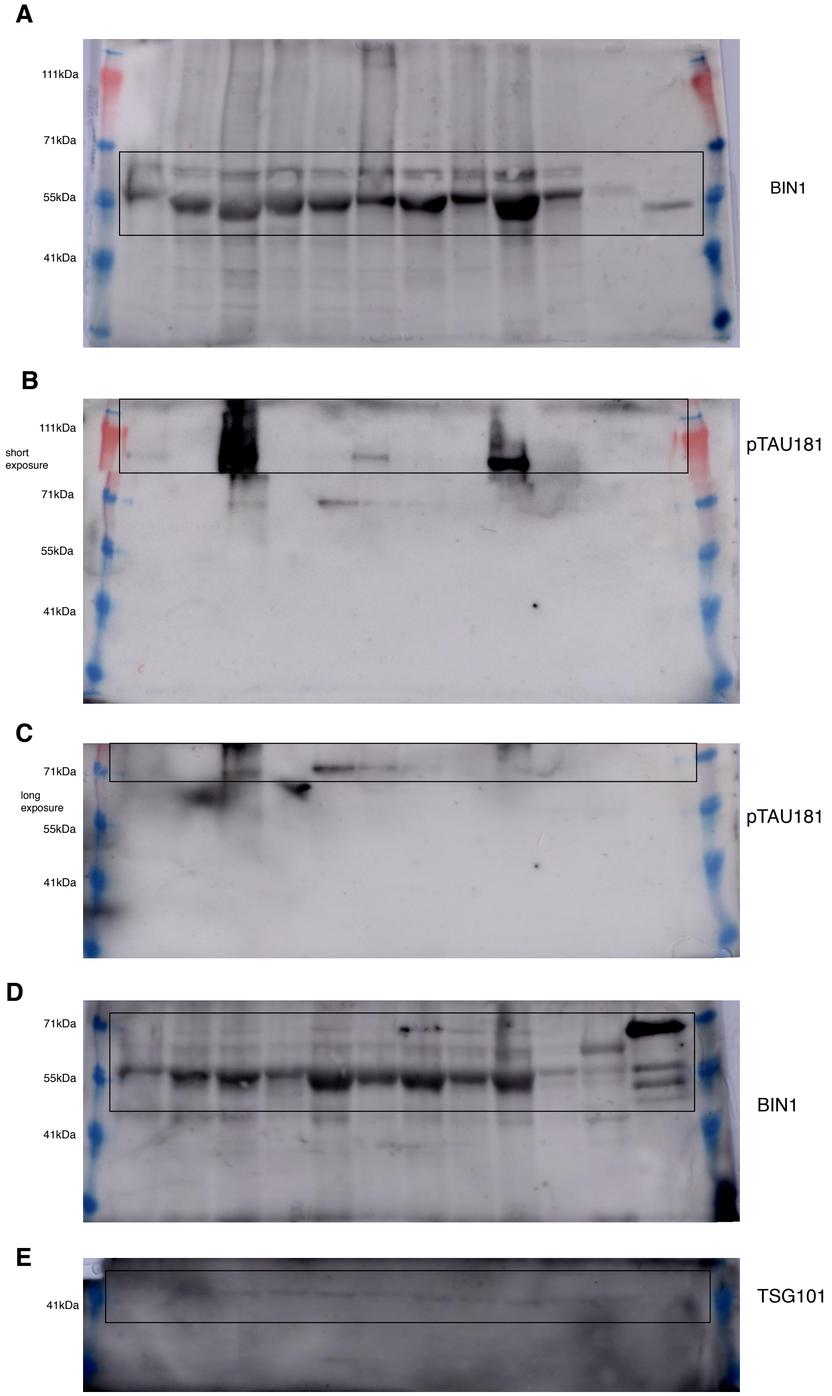

Supplementary Figure 10: Full Western Blots representing images used in Figure 1A and B.

Supplementary Figure 11

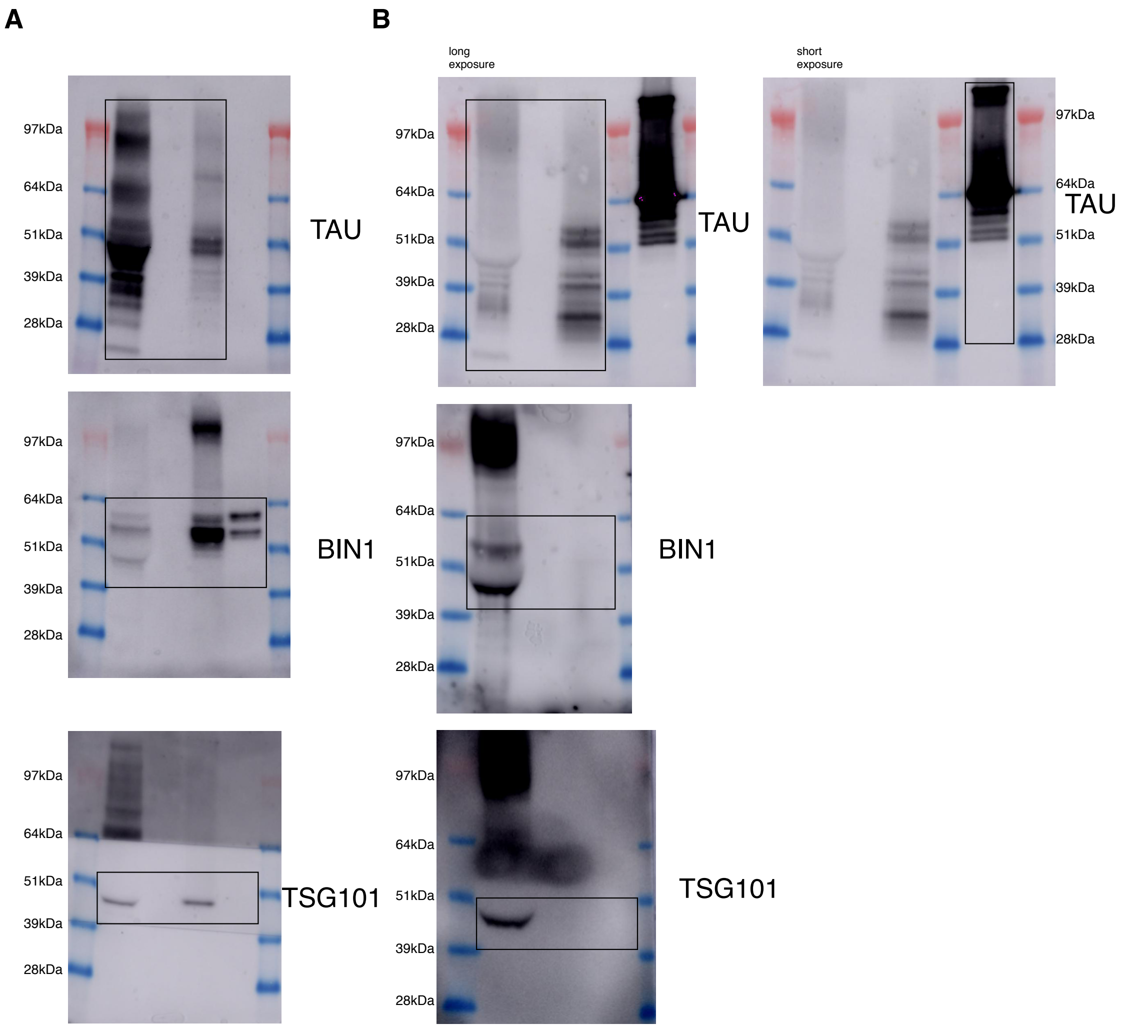

Supplementary Figure 11: Full Western Blots representing images used in Figure 2A and B.

Supplementary Figure 12

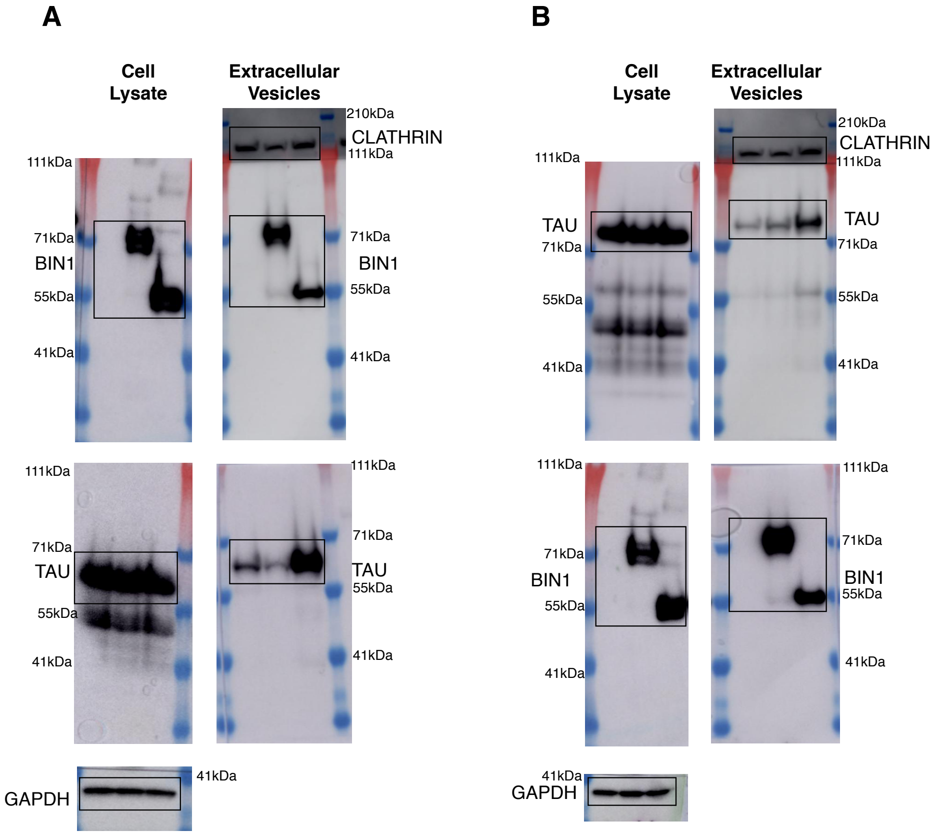

Supplementary Figure 12: Full Western Blots representing images used in Figure 3A and B.

Supplementary Figure 13

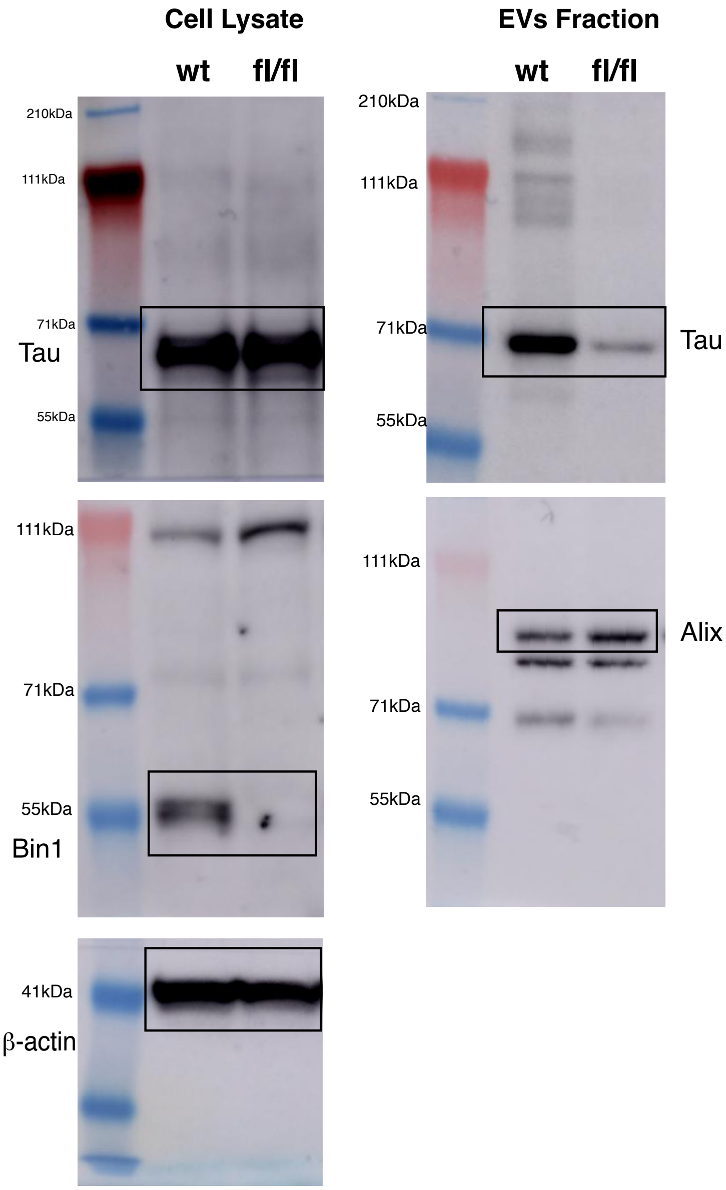

Supplementary Figure 13: Full Western Blots representing images used in Figure 5B.

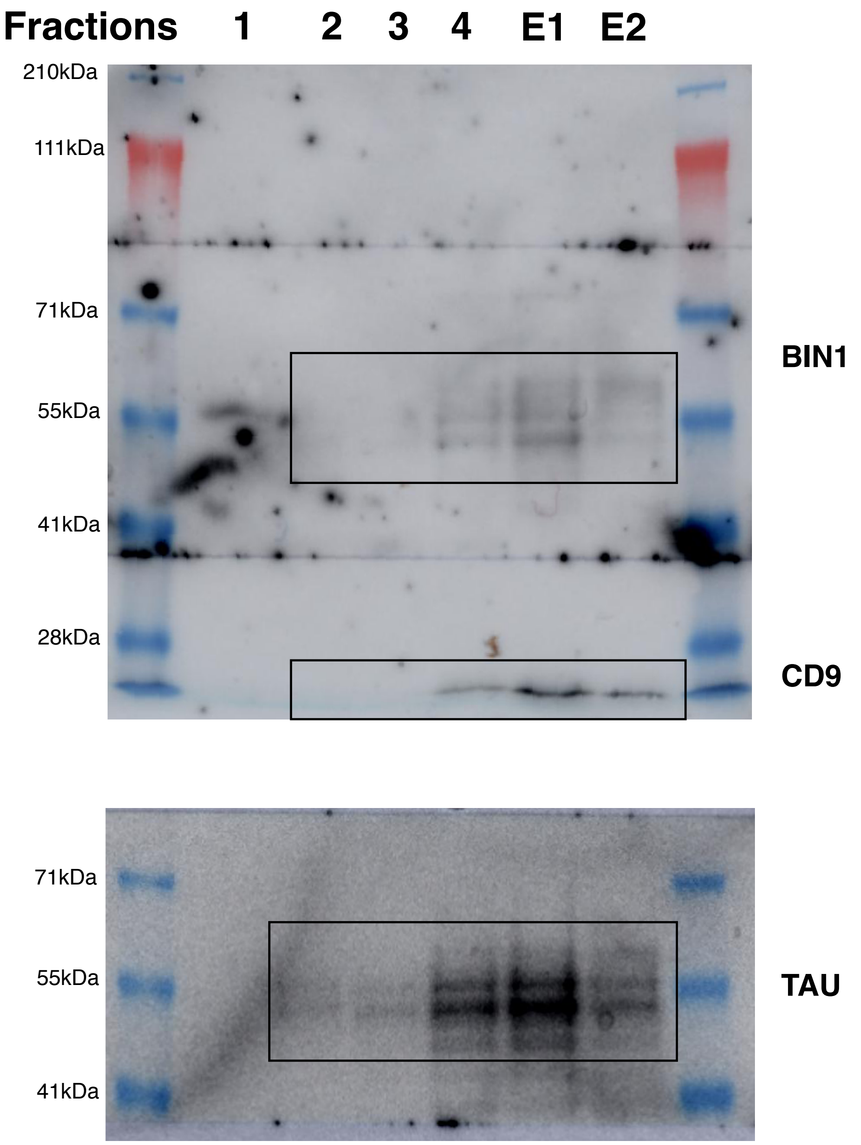

Supplementary Figure 14: Full Western Blots representing images used in Supplementary Figure 1A.

Supplementary Figure 15

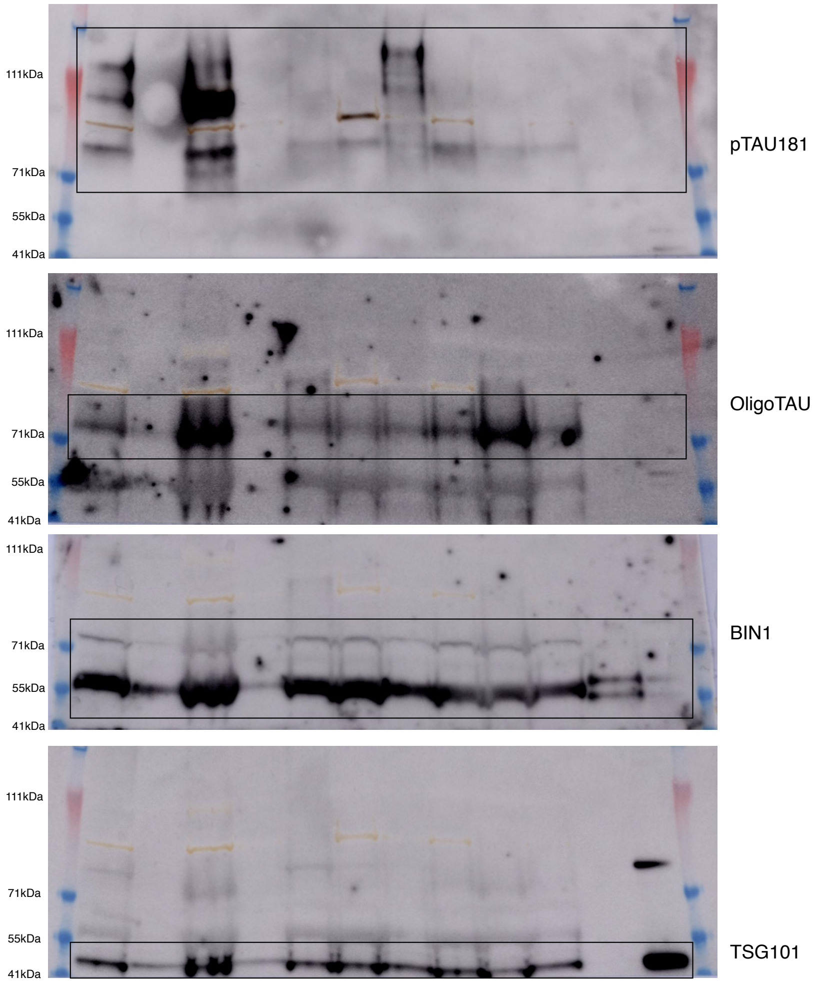

Supplementary Figure 15: Full Western Blots representing images used in Supplementary Figure 2.

Supplementary Figure 16

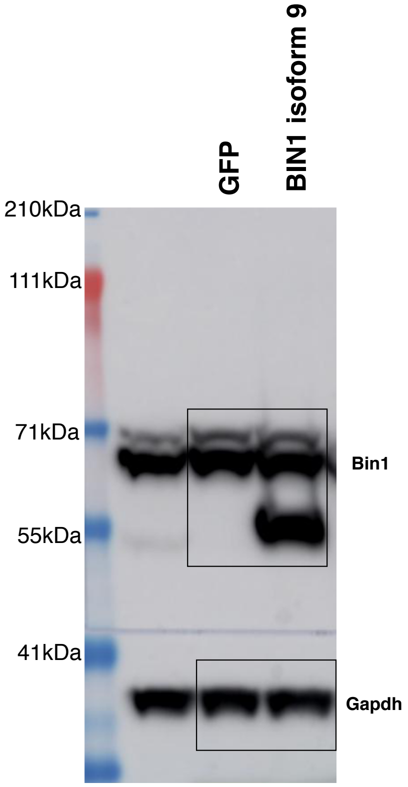

Supplementary Figure 16: Full Western Blots representing images used in Supplementary Figure 4C.

Supplementary Figure 17

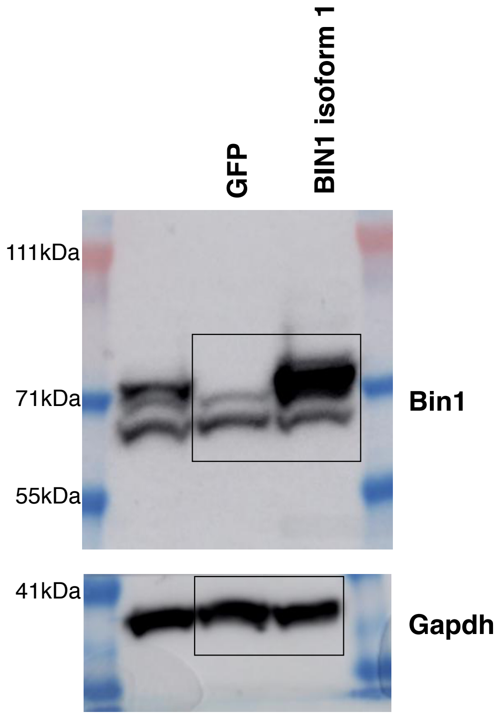

Supplementary Figure 17: Full Western Blots representing images used in Supplementary Figure 6C.

Supplementary Figure 18

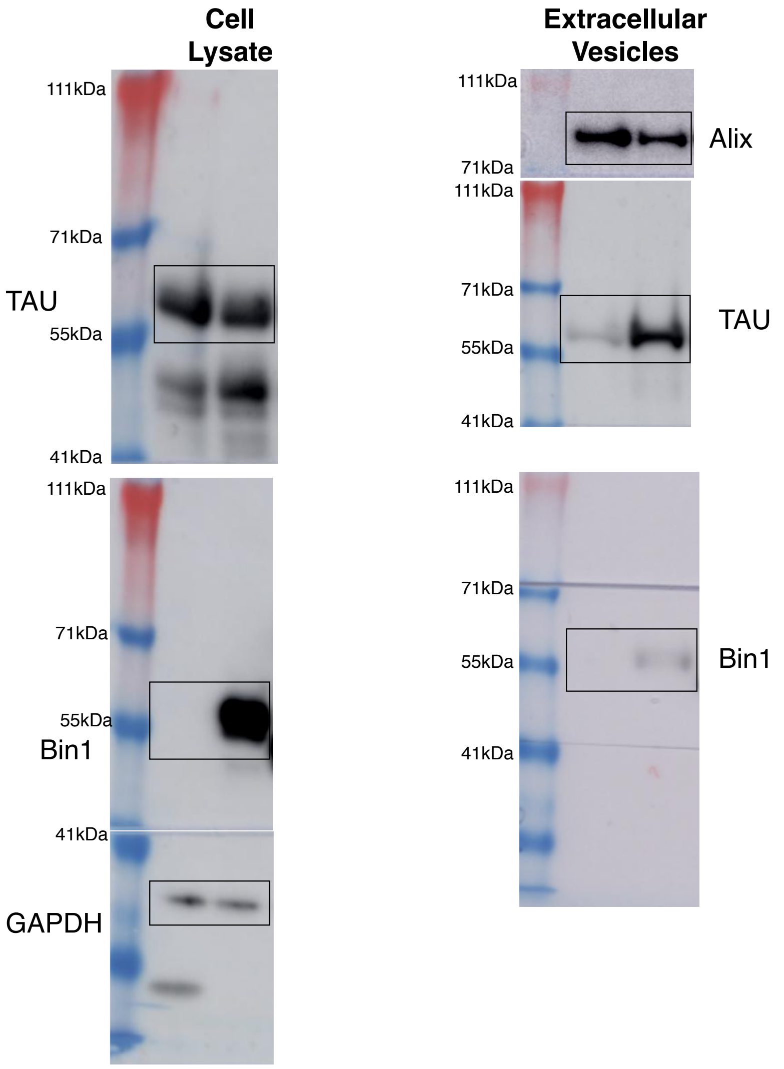

Supplementary Figure 18: Full Western Blots representing images used in Supplementary Figure 8A.

Supplementary Dataset 1: **Demographic, Clinical and Neuropathological Diagnosis of individual donors of Cerebrospinal Fluids samples.** Legend: AD!= Alzheimer disease; CONTR= Control; Amyloid deposit= deposition of amyloid β protein (Aβ) O, A, B, C according to[1]; argyr. grains= Argyrophilic Grain Pathology; Braak Stage= progression of pathological changes in Alzheimer Disease was rated according to Braak's Stages[2]; congoph. Angiopathy= Congophilic Angiopathy; CSF= cerebral spinal fluid; m= male; f= female; n.a.= no data available; NBB = Netherlands Brain Bank number; PMD=post-mortem delay.

Supplementary Dataset 2: **RNAseq gene expression analysis of microglia from $Bin1^{flox/flox}$::Cx3Cr1-Cre^{+}, $Bin1^{wt/flox}$::Cx3Cr1-Cre^{+} and $Bin1^{wt}$ males and females mice at 3 months of age**. Each spreadsheet contains the list of DEGs in different combinations of sex and genotypes: Male Homo vs WT, Male Het vs WT, Female Homo vs WT and Female Het vs WT, respectively.
